# The effect of cardiovascular risk on disease progression in *de novo* Parkinson's disease patients: An observational analysis

**DOI:** 10.3389/fneur.2023.1138546

**Published:** 2023-04-12

**Authors:** Max J. Oosterwegel, Jesse H. Krijthe, Melina G. H. E. den Brok, Lieneke van den Heuvel, Edo Richard, Tom Heskes, Bastiaan R. Bloem, Luc J. W. Evers

**Affiliations:** ^1^Center of Expertise for Parkinson and Movement Disorders, Department of Neurology, Donders Institute for Brain, Cognition and Behaviour, Radboud University Medical Center, Nijmegen, Netherlands; ^2^Department of Data Science, Institute for Computing and Information Sciences, Radboud University, Nijmegen, Netherlands; ^3^Department of Intelligent Systems, Delft University of Technology, Delft, Netherlands; ^4^Department of Neurology, Donders Institute for Brain, Cognition and Behaviour, Radboud University Medical Center, Nijmegen, Netherlands; ^5^Department of Neurology, Amsterdam University Medical Center, Location AMC, Amsterdam, Netherlands

**Keywords:** Parkinson's disease, cardiovascular risk, disease modification, longitudinal modeling, BMI, hypertension, Framingham, causal inference

## Abstract

**Background:**

Currently available treatment options for Parkinson's disease are symptomatic and do not alter the course of the disease. Recent studies have raised the possibility that cardiovascular risk management may slow the progression of the disease.

**Objectives:**

We estimated the effect of baseline cardiovascular risk factors on the progression of Parkinson's disease, using measures for PD-specific motor signs and cognitive functions.

**Methods:**

We used data from 424 *de novo* Parkinson's disease patients and 199 age-matched controls from the observational, multicenter Parkinson's Progression Markers Initiative (PPMI) study, which included follow-up of up to 9 years. The primary outcome was the severity of PD-specific motor signs, assessed with the MDS-UPDRS part III in the “OFF”-state. The secondary outcome was cognitive function, measured with the Montreal Cognitive Assessment, Symbol Digit Modalities Test, and Letter-Number Sequencing task. Exposures of interest were diabetes mellitus, hypertension, body mass index, cardiovascular event history and hypercholesterolemia, and a modified Framingham risk score, measured at baseline. The effect of each of these exposures on disease progression was modeled using linear mixed models, including adjustment for identified confounders. A secondary analysis on the Tracking Parkinson's cohort including 1,841 patients was performed to validate our findings in an independent patient cohort.

**Results:**

Mean age was 61.4 years, and the average follow-up was 5.5 years. We found no statistically significant effect of any individual cardiovascular risk factor on the MDS-UPDRS part III progression (all 95% confidence intervals (CIs) included zero), with one exception: in the PD group, the estimated effect of a one-point increase in body mass index was 0.059 points on the MDS-UPDRS part III per year (95% CI: 0.017 to 0.102). We found no evidence for an effect of any of the exposures on the rate of change in cognitive functioning in the PD group. Similar results were observed for the Tracking Parkinson's cohort (all 95% CIs overlapped with PPMI), but the 95% CI of the effect of body mass index on the MDS-UPDRS part III progression included zero.

**Conclusions:**

Based on this analysis of two large cohorts of *de novo* PD patients, we found no evidence to support clinically relevant effects of cardiovascular risk factors on the clinical progression of Parkinson's disease.

## Introduction

Parkinson's disease (PD) is the second most common neurodegenerative disease after Alzheimer's disease (1, 2). Currently available treatments such as levodopa help to suppress symptoms, but do not influence the underlying pathophysiology (2, 3). A disease-modifying therapy that slows down the progression of the disease, e.g., by attenuating the neurodegeneration, is not available, but recent studies have raised the possibility that management of cardiovascular risk factors may slow the clinical progression of PD (3–9). In the past decades, cardiovascular risk management has led to dramatic reductions in incident cardiovascular disease and stroke (10). Cardiovascular risk factors are also associated with an increased risk for development of other neurodegenerative diseases, including Alzheimer's disease (11). Moreover, risk factors such as hypertension or diabetes mellitus are associated with small vessel disease (12). We hypothesize that this may negatively affect the brain's capability to compensate for disability caused by PD, thereby aggravating its progression over time. Importantly, if this hypothesis is correct, PD patients may experience additional benefits from cardiovascular risk management, and more specifically, benefit from a possible slowing of disease progression.

However, no randomized controlled trial involving cardiovascular risk management in PD has been published and the evidence from observational studies, both cross-sectional and longitudinal, is inconclusive. Using cross-sectional designs, some studies revealed indications of faster PD progression in patients with more cardiovascular risk factors (13–15), while other studies did not find (clear) evidence for these effects (5, 16). Longitudinal studies also offered mixed evidence. Specifically, elevated fasting glucose, hypertension, higher pulse pressure, higher modified Framingham risks score and the presence of white matter hyperintensities were associated with faster cognitive decline in PD (4, 6, 17). Also, hypertension and the presence of coronary artery disease were predictive of faster motor progression in PD patients (4). However, studies on factors such as cholesterol and body mass index (BMI) pointed in opposite directions, with evidence for a protective effect of higher LDL-cholesterol levels and a higher BMI (18–21).^1^

It remains largely unclear whether the described effects are PD-specific, or whether these can be explained by the effects of cardiovascular risk factors in the general population. There are two reasons for this. First, most studies did not include a non-PD control group, or only a control group with a small sample size (typically < 100 subjects), leaving the question if there is a significant interaction between cardiovascular risk and PD largely unanswered. Second, longitudinal studies have mostly focused on measuring disease progression using cognitive assessments. More insights into the effects on the progression of more PD-specific motor signs would help to determine whether cardiovascular risk management has PD-specific effects. In addition, studies that included a control group and motor assessments have focused on establishing predictive relationships (4). It remains unclear whether the observed relationships remain when appropriately adjusted for confounders, and whether an intervention such as cardiovascular risk management can modify the disease trajectory of PD patients.

The aim of this study was to assess the effects of cardiovascular risk factors on progression of motor PD-specific motor signs and cognitive functioning in *de novo* PD patients, using longitudinal, observational data from both PD patients and controls. To appropriately adjust for confounding, we based our analysis on causal models represented by directed acyclic graphs (DAGs), which we developed based on the literature and input from clinical experts.

## Methods

### Datasets

We used observational data from the Parkinson's Progression Markers Initiative (PPMI) for our primary analysis, and validated our findings where possible using the Tracking Parkinson's cohort (see below for details). PPMI is an observational, longitudinal, multicenter study designed to collect clinical, imaging, and biosample data to define biomarkers of PD progression. Each participating PPMI site received ethical approval before study initiation and obtained written informed consent from all participants. Detailed information is available from the PPMI study publication (22).

In brief, individuals were recruited *via* twenty-one clinical sites, sixteen in the US and five in Europe. Recruitment started in 2010 and the study is still ongoing. The main recruitment strategy was based on referral by physicians. Main inclusion criteria for the PD group were: (1) presence of an asymmetric resting tremor, asymmetric bradykinesia or two of bradykinesia, resting tremor and rigidity; (2) diagnosis of PD for 2 years or less at screening, and not expected to require PD medication within 6 months from the start of the study; (3) deficit consistent with PD on SPECT imaging. Inclusion criteria for the control group consisted of: (1) no current or active clinically significant neurological disorder; (2) no significant cognitive impairment (Montreal Cognitive Assessment score of higher than twenty-six); (3) no first-degree relatives with PD. Our rationale for including a control group was to ascertain that any observed effect of the studied cardiovascular risk factors was PD-specific.

Exclusion criteria for both groups related to the safe execution of all study procedures such as the lumbar puncture. After enrollment, participants were assessed every 3 months during the first year and every 6 months thereafter, up to 9 years. In the current analyses, we use data of all follow-up visits included in the July 28th 2020 version of the data from the website (www.ppmi-info.org). Subjects were included in the analysis when data on the variables of interest was available.

In order to validate our findings in an independent cohort, we performed a secondary analysis using data from the Tracking Parkinson's cohort (23). Tracking Parkinson's is a multicenter cohort study including 2,247 PD patients (1987 recent onset, 260 young onset). Patients were recruited *via* 72 sites in the UK that provide secondary care to PD patients. In contrast with PPMI, both drug-naive patients and treated patients were included. Individuals were assessed every 18 months from baseline. We accessed this data *via* the Critical Path for Parkinson's (CPP) database (24), which contained the recent onset cases (maximum of 3 years since PD diagnosis) and their assessments up to 36 months.

### Outcome measures

The predefined primary outcome measure consisted of the MDS-UPDRS part III motor scores (25). We considered all measurements taken in the “OFF-state”, defined as no medication intake for at least 6 h since the last intake of levodopa/dopamine agonist while acknowledging that not all effects have worn off after 6 h. Secondary outcome measures related to progression in the cognitive domain. The Montreal Cognitive Assessment (MoCA) was used as a test for general cognitive functioning, while the Symbol Digit Modalities Test (SDMT) and Letter-Number Sequencing (LNS) test were used to specifically assess the effect on executive functioning.

### Cardiovascular risk factors

Exposures of interest were measured at baseline and included the presence of diabetes mellitus type II, hypertension, adiposity, hypercholesterolemia, a history of cardiovascular events and overall cardiovascular risk. The exposures were operationalized according to the following definitions. A diagnosis of diabetes mellitus type II was extracted from the medical history. Hypertension was defined as a systolic blood pressure of ≥140 mmHg or a medical history including terms for hypertension (e.g., high blood pressure). Similarly, the presence of hypercholesterolemia was based on the medical history and lipid measurements; subjects were labeled as having hypercholesterolemia if LDL cholesterol levels were above 100 mg/dL, if total cholesterol levels exceeded 180 mg/dL, if HDL cholesterol levels were below 40 mg/dL for men or below 50 mg/dL for women, or if the medical history included terms for hypercholesterolemia (e.g., high cholesterol). These thresholds were based on current guidelines of cardiovascular disease risk assessment (26). The degree of adiposity was assessed using BMI. A history of cardiovascular events served as proxy for increased cardiovascular risk, and was defined as a medical history of coronary artery disease (myocardial infarction or angina pectoris) and cerebrovascular disease (cerebrovascular accident or transient ischemic attack). Furthermore, overall cardiovascular risk was assessed using a modified version of the Framingham risk score. This score takes a combination of cardiovascular risk factors into account to estimate the 10-year cardiovascular disease risk of an individual, using age, sex, BMI, diagnosis of diabetes mellitus, systolic blood pressure and use of antihypertensive medication (6, 27). Smoking status was not considered since this was not recorded in the PPMI dataset.

### DAGs

Directed acyclic graphs (DAGs) for each identified cardiovascular risk factor were constructed based on the literature and input from clinical experts, consisting of a neurologist specialized in movement disorders, a neurologist specialized in vascular disease, a dietician specialized in PD, and a clinical researcher specialized in PD. DAGs reflect the presumed relationships between variables, and make the assumptions underlying the analyses explicit (and thus transparent). Moreover, they are used to find a minimal sufficient adjustment set to adjust for confounding factors (28). In our constructed DAGs, the individual cardiovascular risk factors were the exposure (measured at baseline), and a measure of PD progression such as MDS-UPDRS part III was the outcome. All confounders were also measured at baseline.

**Figure 1:**
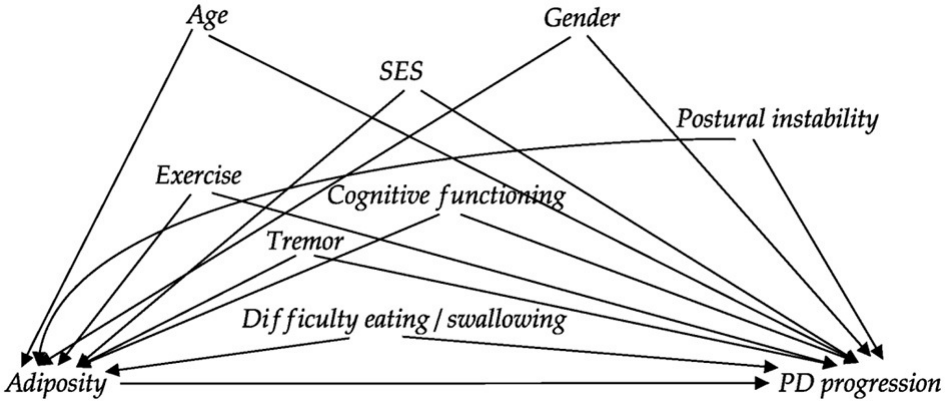
Directed acyclic graph (DAG) reflecting the assumed relationships between variables for the analysis of the effect of adiposity on PD progression. Relationships between confounders are not shown to avoid clutter (and because these did not alter the required adjustment set). The DAGs were constructed together with multiple clinical PD experts. DAGs for the other exposures are displayed in the supplement. SES: socioeconomic status.

Figure 1 displays the DAG for adiposity; the constructed DAGs for all exposures can be found in the supplement. A minimal sufficient adjustment set was derived from these DAGs by applying the d-separation criterion (29). To estimate the effect of hypertension, hypercholesterolemia, and diabetes mellitus, adjustments for adiposity, sex, age and socioeconomic status were required. The models for cardiovascular event history and cardiovascular risk score had to include adjustments for socioeconomic status, sex and age. The relationship between adiposity and progression of PD is relatively complex, because various PD-specific factors may influence the weight of individual PD patients. Besides age, gender and socioeconomic status, we identified cognitive functioning, tremor level, swallowing/eating problems and postural instability as the most important potential confounders for the causal relationship between adiposity and disease progression (30–33).

We operationalized the variables in the adjustment sets according to the following definitions. Years of education was used as proxy for socioeconomic status and BMI as measure of adiposity. We quantified postural instability as the sum of four items from the MDS-UPDRS (items 2.12, 2.13, 3.10, and 3.12). Cognitive functioning was quantified using the MoCA total score, and we used item 2.10 from the MDS-UPDRS as a measure of tremor. Lastly, swallowing and eating problems were rated as the sum of MDS-UPDRS items 2.3 and 2.4.

### Statistical analysis

We used linear mixed models to estimate the effect of baseline cardiovascular risk factors on PD progression over time. For each cardiovascular risk factor, outcome measure and group (PD and control), a separate model was estimated, to be able to account for the different adjustment sets. We used a random intercept and random slope for the time variable per individual. For the PD group, time was defined as years since diagnosis to correct for disease duration. For the control group, years since enrollment was used.

Besides the exposure of interest and the time variable, each model contained the adjustment sets, derived from the constructed DAGs. We added the baseline score of the outcome measure to this set of confounders to correct for regression to the mean. Each variable in the model included an interaction with the time variable, because we were interested in its effect on progression (i.e., rate of change over time). The unadjusted models used the same time variable and the same random effects structure but did not correct for the identified confounders.

Clinically relevant effects of the estimated coefficients for the rate of change (betas) were defined for our primary outcome measure by combining the minimally clinically important difference (MCID) on the MDS-UPDRS part III of three points with a reasonable time period (5 years) and a realistic intervention on the exposure (32, 34). Specifically, based on the literature we assumed a weight loss of 10% (35). With a mean BMI of 27.1 in the PPMI cohort an intervention on BMI would amount to a BMI loss of 2.71 on average. If we want this intervention to reach the MCID threshold within 5 years BMI should contribute to 0.22 extra motor points per BMI point per year. Similarly, we assumed a three-point reduction in the modified Framingham risk score to be realistic. For the dichotomous exposures, we assumed a change from the risk factor being present to absent.

All models were implemented in R 3.6.3 using the nlme library (31, 32). We used the optim optimizer with a maximum of 100 iterations. The variance-covariance matrices $R_{i}$ and D were not changed from their defaults; the matrix for random effects D was unconstrained, and the residual variance covariance matrix $R_{i}$ implied independent and homoscedastic residual errors.

## Results

**Figure 2:**
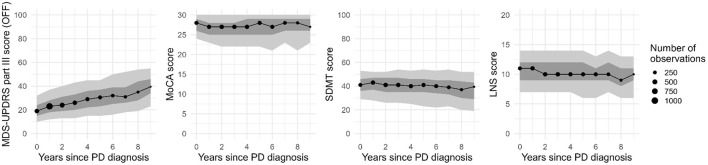
Progression of the outcome measures on the group level for the PD cohort from the PPMI study (median, 30th and 70th percentiles in dark gray, and 10th and 90th percentiles in light gray; missing values were excluded). Only years with at least 30 assessments are shown. MDS-UPDRS, Movement Disorders Society Unified Parkinson Disease Rating Scale; MoCA, Montreal Cognitive Assessment; SDMT, Symbol Digit Modalities Test; LNS, Letter-Number Sequencing task.

We included 424 PD patients and 199 controls from the PPMI dataset. For each analysis, only the subjects with available data on the baseline covariates of the model were used. Mean follow-up was 5.5 years (SD: 2.5 years, maximum of 9 years). The average number of motor measurements taken during the study was 8.2 for the PD individuals (SD: 3.4) and 6.4 for controls (SD: 2.0). Additionally, on average 6.6 MoCA, LNS, and SDMT assessments (SD: 2.2) were available per individual. Baseline characteristics of the included individuals are shown in Table 1. The observed progression of the different outcome measures is displayed in Figure 2. Characteristics of the Tracking Parkinson's cohort can be found in Supplementary Table 4, Supplementary Figure 2.

**Table 1:** Demographics, disease characteristics and cardiovascular risk factors at baseline of individuals included in the PPMI analyses.

	**PD (*N* = 424)**	**Non-PD control (*N* = 199)**	***p*-value**
**Age**			
Mean (SD)	61.7 (9.71)	60.8 (11.2)	0.37
**Sex**			
Men	277 (65.3%)	126 (63.3%)	0.84
**Education (years)**			
Mean (SD)	15.6 (2.96)	16.0 (2.92)	0.09
**Disease duration (years)**			
Mean (SD)	0.55 (0.54)		
CV event history	22 (5.2%)	6 (3.0%)	0.31
Hypertension	197 (46.5%)	91 (45.7%)	0.93
Hypercholesterolemia	203 (47.9%)	109 (54.8%)	0.13
Diabetes	21 (5.0%)	9 (4.5%)	0.97
Statins	133 (31.4%)	62 (31.2%)	0.94
Antihypertensives	172 (40.6%)	90 (45.2%)	0.19
**Modified Framingham risk score**			
Mean (SD)	13.0 (4.72)	13.1 (5.19)	0.87
**Systolic blood pressure (mmHg)**			
Mean (SD)	131 (17.1)	132 (17.2)	0.40
**Total cholesterol (mg/dl)**			
Mean (SD)	184 (48.4)	186 (40.3)	0.84
Missing	349 (82.3%)	114 (57.3%)	
**HDL cholesterol (mg/dl)**			
Mean (SD)	56.6 (18.9)	56.1 (17.4)	0.85
Missing	270 (63.7%)	99 (49.7%)	
**LDL cholesterol (mg/dl)**			
Mean (SD)	109 (33.1)	115 (28.6)	0.44
Missing	345 (81.4%)	184 (92.5%)	
**Body mass index (kg/m** ^2^ **)**			
Mean (SD)	27.1 (4.64)	26.9 (4.42)	0.60
**MDS-UPDRS part II**			
Mean (SD)	5.90 (4.19)	0.46 (1.02)	< 0.001
**MDS-UPDRS part III**			
Mean (SD)	20.9 (8.84)	1.21 (2.19)	< 0.001
**MoCA**			
Mean (SD)	27.1 (2.32)	28.2 (1.11)	< 0.001
**LNS**			
Mean (SD)	10.6 (2.65)	10.9 (2.57)	0.21
**SDMT**			
Mean (SD)	41.2 (9.71)	46.8 (10.5)	< 0.001

MoCA results from the screening visit (maximum of 30 days prior to baseline) are shown as MoCA was not measured at baseline. CV, cardiovascular; HDL, high-density lipoprotein; LDL, low-density lipoprotein; PD, Parkinson's disease; MoCA, Montreal Cognitive Assessment; LNS, Letter-Number Sequencing; SDMT, Symbol Digit Modalities Test; MDS-UPDRS, Movement Disorders Society Unified Parkinson Disease Rating Scale.

**Figure 3:**
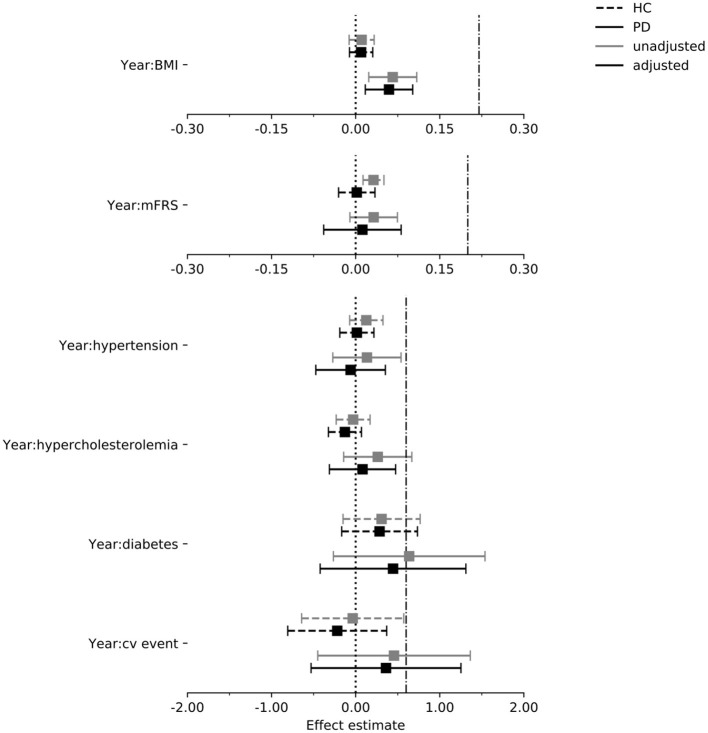
Estimated effect of each exposure on the rate of change in the MDS-UPDRS part III score per year in the PPMI cohort. Dash dot line indicates threshold for assumed clinically relevant effect (see “Methods”). Intervals are 95% confidence intervals. mFrs, modified Framingham risk score; BMI, body mass index; cv, cardiovascular; HC, healthy control; PD, Parkinson's disease.

Figure 3 shows the estimated effect of each exposure on the rate of change of the MDS-UPDRS part III score in the PPMI cohort. The 95% confidence intervals (CIs) for the estimated effects of all individual cardiovascular risk factors included zero in both the PD and control group, with one exception; in the PD group, the estimated effect of one point increase on the BMI was 0.059 points on the MDS-UPDRS part III per year (95% CI: 0.017–0.102). However, the 95% CI does not reach the assumed clinically relevant effect size of 0.22. The effect of BMI was not observed in the control group (beta = 0.01, [95% CI: −0.011 to 0.030]).

**Figure 4:**
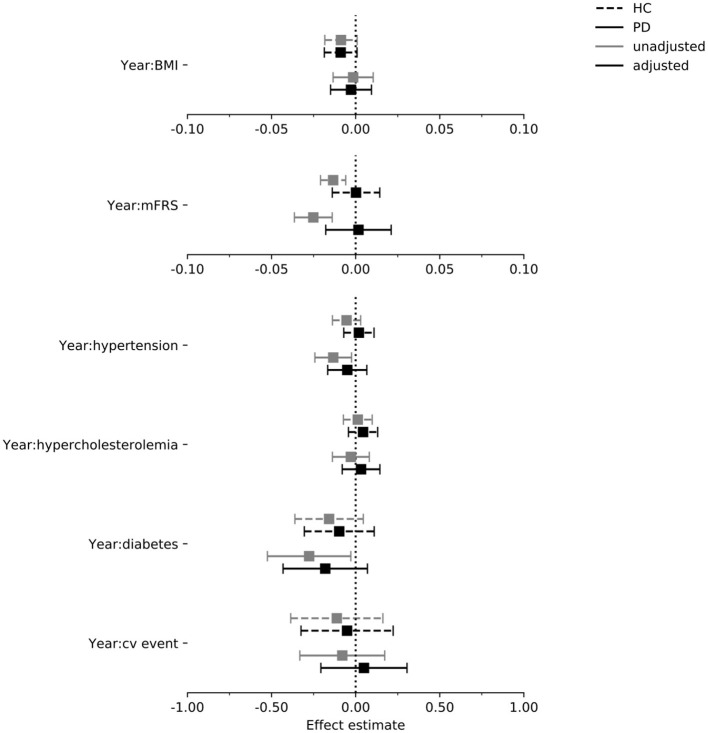
Estimated effect of each exposure on the rate of change in MoCA per year in the PPMI cohort. Intervals are 95% confidence intervals. mFrs, modified Framingham risk score; BMI, body mass index; cv, cardiovascular; HC, healthy control; PD, Parkinson's disease.

**Figure 5:**
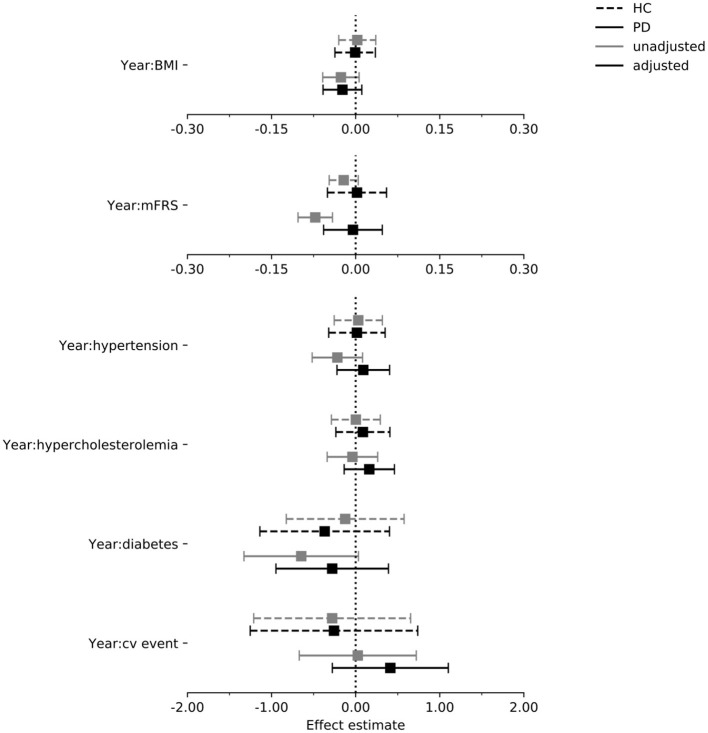
Estimated effect of each exposure on the rate of change in the SDMT score per year in the PPMI cohort. Intervals are 95% confidence intervals. mFrs, modified Framingham risk score; BMI, body mass index; cv, cardiovascular; HC, healthy control; PD, Parkinson's disease.

**Figure 6:**
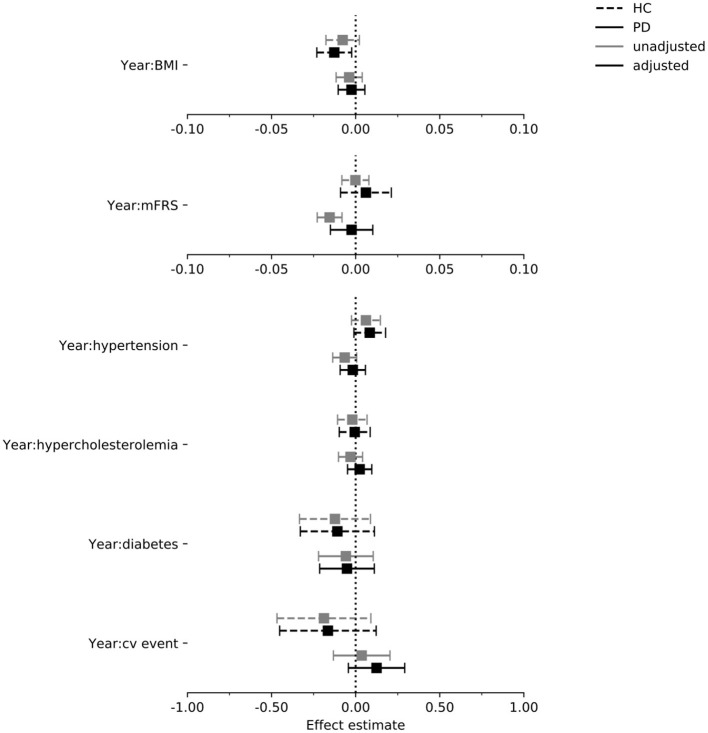
Estimated effect of each exposure on the rate of change in the LNS score per year in the PPMI cohort. Intervals are 95% confidence intervals. mFrs, modified Framingham risk score; BMI, body mass index; cv, cardiovascular; HC, healthy control; PD, Parkinson's disease.

In Figures 4–6 we display the results for the cognitive outcome measures (i.e., MoCA, SDMT and LNS). In the unadjusted models of cognitive functioning in the PD group, confidence intervals of the modified Framingham risk score (MoCA: beta = −0.025, [95% CI −0.036 to −0.014], SDMT: beta = −0.072, [95% CI −0.103 to −0.041], LNS: beta = −0.015, [95% CI −0.023 to −0.008]), the presence of diabetes (MoCA: beta = −0.277, [95% CI −0.525 to −0.029]), and the presence of hypertension (MoCA: beta = −0.133, [95% CI −0.242 to −0.024]) indicated faster deterioration. However, for none of the exposures there was evidence for an effect on the rate of change in cognitive functioning in the PD group when the confounders were taken into account.

We used data from the Tracking Parkinson's cohort to verify the estimated effects on the MDS-UPDRS part III and MoCA in the PD group (this cohort did not include a control group, and SDMT and LNS data was not available). All 95% CIs of the Tracking Parkinson's overlapped with the 95% CIs of PPMI, both for the unadjusted and adjusted estimates. However, in contrast to PPMI the adjusted 95% CI for the effect of BMI on the MDS-UPDRS part III included zero in the Tracking Parkinson's cohort (beta = 0.018, [95% CI −0.033 to 0.070]). Exact results for each exposure in each model and figures comparing the results of PPMI and Tracking Parkinson's can be found in Supplementary Figures 3, 4, Supplementary Tables 3, 5.

Model diagnostics were deemed sufficient: normality and homogeneity of the residuals was not severely violated and there was no clear evidence of non-linearity of one of the models (see Supplementary material).

## Discussion

Based on careful analysis of two large cohort studies (PPMI and Tracking Parkinson's), we found no evidence for clinically relevant effects of hypertension, BMI, hypercholesterolemia, diabetes mellitus or prior cardiovascular events on the progression of motor and cognitive impairments in PD. After adjustments for confounding, only BMI showed a statistically significant effect on the rate of change in “OFF”-state motor functioning, but this effect was not reproduced in an independent cohort. Note that the analysis of the effects of diabetes mellitus and prior cardiovascular events was limited by the small number of cases, which is also reflected by the large confidence intervals. Therefore, contrary to hypertension, BMI, hypercholesterolemia, we cannot rule out a clinically relevant effect of diabetes mellitus and prior cardiovascular events.

These findings diverge from a few other longitudinal studies that reported accelerated cognitive decline in PD patients with a higher cardiovascular risk score and a history of hypertension, and a protective effect of higher LDL cholesterol and BMI (6, 17, 18, 20, 21). Several factors may explain these differences. First, we defined a more restrictive set of outcome measures a priori, reducing the risk of false positives. Second, the differences between our unadjusted and adjusted estimates, particularly for cognitive functioning, demonstrate the importance of carefully considering confounding. There are notable differences between studies in how adjustments were performed. For example, Chahine et al. adjusted for the effects of age on baseline scores, but not for its effects on the rate of change (6). In the supplement, we illustrate the impact of such differences by showing that an effect of the modified Framingham risk score on PD progression disappears when the effect of age on the rate of change is taken into account. Importantly, our effect estimates for BMI, hypertension and diabetes on motor progression were within the 95% confidence intervals of the effect estimates from the DeNoPa cohort study (4) (both adjusted and unadjusted estimates), and the results for BMI are similar to another longitudinal analysis of the PPMI cohort (19), which increases the confidence in our findings.

Considering our hypothesis of small vessel disease resulting in faster disease progression, we expected that multiple cardiovascular risk factors would show an effect on PD progression. This does not match with our finding that only BMI showed a statistically significant effect. Therefore, other mechanisms than small vessel disease might be more likely to explain a potential effect of BMI on the progression of PD-specific motor signs. As indicated by our DAG, the effect of BMI might still be confounded by exercise and its beneficial effect on motor functioning (36). Therefore, accurate measures of physical activity would be a valuable addition to such cohort studies.

The strengths of this study included the large number of measurements used to estimate the progression rate, relatively long follow-up (mean of 5.5 years), the inclusion of a control group, and the validation of our findings in an independent study cohort. Moreover, we made the assumptions underlying our causal analysis explicit and transparent using DAGs, which were carefully constructed together with multiple clinical PD experts. Although randomized controlled trials remain the gold standard to evaluate the effect of interventions, the increasing amount of observational data, when combined with appropriate causal analyses, forms a valuable additional source of evidence. This is particularly important since conducting an RCT (randomized controlled trial) is expensive and comes with ethical challenges, for example around withholding treatments that are known to be effective (as is the case for cardiovascular risk management in terms of benefits not specific to PD).

Our study was not without limitations. First, assigning a causal meaning to our effect estimates depends on whether the DAGs are a reasonably accurate representation of reality. Despite our efforts to carefully construct the DAGs, it may not contain all sources of confounding. For example, exercise is a plausible confounder that was not included in the models, because the included studies did not collect such information at baseline (36). This means that a potential effect of exercise might now be (partly) attributed to cardiovascular risk factors instead. Other factors that are very difficult to test for, but that are possible confounders, include the genetic profile, unknown comorbidities and lifestyle-related variables like stress and diet. Second, our variables may not accurately measure all constructs we are interested in. For example, BMI is not a comprehensive measure of adiposity, which is ideally measured with a vector of variables including weight, height, body fat and waist circumference. A variable closer to the construct or closer to a well-defined intervention could tell us more about the possible mechanisms involved (37). Additionally, baseline blood pressure measurements were used in our analyses, but these single measurements do not reflect the long-term exposure to this risk factor. In other neurodegenerative diseases such as dementia, the association with blood pressure is complex and age dependent: hypertension in midlife and decreasing blood pressure in later life are associated with an increased risk for dementia (11, 38, 39). Third, strictly speaking our results on the effect of cardiovascular risk management relate to treatment before PD diagnosis or early in the disease course, because we modeled the effect of cardiovascular risk measured at baseline in a cohort with recent onset PD. Future research can obtain a more complete answer by explicitly modeling the exposures and their confounders over time instead of the risk factors at baseline (40). Finally, although our analysis was based on a relatively long follow-up, demonstrating the effects of cardiovascular risk factors may require even longer follow-up, following the example of studies such as the Framingham Heart Study (41). This might also explain why we did not observe known effects of cardiovascular risk factors on cognition in the control group using the MoCA (42). Future research using data with longer follow-up will also allow for the inclusion of functional outcomes such as time to nursing home admission and time to first fall.

In conclusion, our analysis of two large cohorts of *de novo* PD patients indicates that it is unlikely that important cardiovascular risk factors (i.e., BMI, hypertension and hypercholesterolemia) have clinically relevant effects on the clinical progression of PD within 5 years. Modeling the risk factors and their confounders over longer time periods, as well as modeling more explicit interventions, can complement this line of work to obtain a more complete picture of the effect of cardiovascular risk management in PD.

## Data availability statement

The data analyzed in this study were obtained from the Parkinson's Progression Markers Initiative (https://www.ppmi-info.org/access-data-specimens/download-data) and the Tracking Parkinson's study via the Critical Path for Parkinson's (CPP) Integrated Parkinson's Database (https://c-path.org/programs/cpp/). The following licenses/restrictions apply: Investigators seeking access to PPMI data must sign the Data Use Agreement, submit an Online Application and comply with the study Publications Policy (via https://www.ppmi-info.org/access-data-specimens/download-data). Investigators seeking access to Tracking Parkinson's study data must apply via the Tracking Parkinson's website (https://www.trackingparkinsons.org.uk/about-1/data/) or the Critical Path for Parkinson's (CPP) Integrated Parkinson's Database (https://c-path.org/programs/cpp/).

## Author contributions

MO: execution of research project, design and execution of statistical analysis, and writing first draft. JK and LE: conception of research project, design, review and critique regarding statistical analysis, and review of manuscript. MB: execution of research project and review of manuscript. LH and ER: conception of research project and review of manuscript. TH: conception and organization of research project, review and critique regarding statistical analysis, and review of manuscript. BB: conception and organization of research project and review of manuscript.

## Footnotes

^1^A list of studies on cardiovascular risk factors and Parkinson's disease progression and their most relevant results can be found in Supplementary Tables 1, 2.

## References

[1] von Campenhausen S Bornschein B Wick R Bötzel K Sampaio C Poewe W. Prevalence and incidence of Parkinson's disease in Europe. Europ Neuropsychopharmacol. (2005) 15:473–90. 10.1016/j.euroneuro.2005.04.007 15963700

[2] Verschuur CV Suwijn SR Boel JA Post B Bloem BR van Hilten JJ. Randomized delayed-start trial of levodopa in Parkinson's disease. New England J Med. (2019) 380:315–24. 10.1056/NEJMoa1809983 30673543

[3] Lang AE Espay AJ. Disease modification in Parkinson's disease: current approaches, challenges, and future considerations. Movement Disorders. (2018) 33:660–77. 10.1002/mds.27360 29644751

[4] Mollenhauer B Zimmermann J Sixel-Döring F Focke NK Wicke T Ebentheuer J. Baseline predictors for progression 4 years after Parkinson's disease diagnosis in the De Novo Parkinson Cohort (DeNoPa). Movement Disorders. (2019) 34:67–77. 10.1002/mds.27492 30468694

[5] Malek N Lawton MA Swallow DM Grosset KA Marrinan SL Bajaj N. Vascular disease and vascular risk factors in relation to motor features and cognition in early Parkinson's disease. Movement Disorders. (2016) 31:1518–26. 10.1002/mds.26698 27324570 PMC5082556

[6] Chahine L Dos Santos C Fullard M Scordia C Weintraub D Erus G. Modifiable vascular risk factors, white matter disease and cognition in early Parkinson's disease. Europ. J. Neurol. (2019) 26:246–e18. 10.1111/ene.13797 30169897 PMC6329649

[7] Bohnen NI Albin RL. White matter lesions in Parkinson disease. Nat. Rev. Neurol. (2011) 7:229. 10.1038/nrneurol.2011.21 21343896 PMC3739056

[8] Potashkin J Huang X Becker C Chen H Foltynie T Marras C. Understanding the links between cardiovascular disease and Parkinson's disease. Movement Disorders. (2020) 35:55–74. 10.1002/mds.27836 31483535 PMC6981000

[9] Stojkovic T Stefanova E Soldatovic I Markovic V Stankovic I Petrovic I. Exploring the relationship between motor impairment, vascular burden and cognition in Parkinson's disease. J Neurol. (2018) 265:1320–7. 10.1007/s00415-018-8838-3 29572571

[10] World Health Organization. Prevention of Cardiovascular Disease: Guidelines for Assessment and Management of Total Cardiovascular Risk. Geneva: World Health Organization (2007).

[11] Livingston G Huntley J Sommerlad A Ames D Ballard C Banerjee S. Dementia prevention, intervention, and care: 2020 report of the lancet commission. Lancet. (2020) 396:413–46. 10.1016/S0140-6736(20)30367-6 32738937 PMC7392084

[12] Pantoni L. Cerebral small vessel disease: from pathogenesis and clinical characteristics to therapeutic challenges. Lancet Neurol. (2010) 9:689–701. 10.1016/S1474-4422(10)70104-6 20610345

[13] Jones J Malaty I Price C Okun M Bowers D. Health comorbidities and cognition in 1948 patients with idiopathic Parkinson's disease. Parkinsonism Relat Disord. (2012) 18:1073–8. 10.1016/j.parkreldis.2012.06.004 22776043 PMC6545886

[14] Swallow DM Lawton MA Grosset KA Malek N Klein J Baig F. Statins are underused in recent-onset Parkinson's disease with increased vascular risk: findings from the UK Tracking Parkinson's and Oxford Parkinson's Disease Centre (OPDC) discovery cohorts. J Neurol Neurosurg Psychiatry. (2016) 87:1183–90. 10.1136/jnnp-2016-313642 27671901 PMC5116532

[15] Papapetropoulos S Ellul J Argyriou A Talelli P Chroni E Papapetropoulos T. The effect of vascular disease on late onset Parkinson's disease. Europ J Neurol. (2004) 11:231–5. 10.1046/j.1468-1331.2003.00748.x 15061824

[16] Kotagal V Albin RL Müller ML Koeppe RA Frey KA Bohnen NI. Modifiable cardiovascular risk factors and axial motor impairments in Parkinson disease. Neurology. (2014) 82:1514–20. 10.1212/WNL.0000000000000356 24682965 PMC4011463

[17] Doiron M Langlois M Dupré N Simard M. The influence of vascular risk factors on cognitive function in early Parkinson's disease. Int J Geriatr Psychiatry. (2018) 33:288–97. 10.1002/gps.4735 28509343

[18] Yoo HS Chung SJ Lee PH Sohn YH Kang SY. The influence of body mass index at diagnosis on cognitive decline in Parkinson's disease. J Clinical Neurology. (2019) 15:517–26. 10.3988/jcn.2019.15.4.517 31591841 PMC6785479

[19] Kim R Jun J-S. Impact of overweight and obesity on functional and clinical outcomes of early Parkinson's disease. J Am Med Direct Associat. (2020) 21:697–700. 10.1016/j.jamda.2019.11.019 31928933

[20] Sterling NW Lichtenstein M Lee E-Y Lewis MM Evans A Eslinger PJ. Higher plasma LDL-cholesterol is associated with preserved executive and fine motor functions in Parkinson's disease. Aging Dis. (2016) 7:237. 10.14336/AD.2015.1030 27330838 PMC4898920

[21] Huang X Auinger P Eberly S Oakes D Schwarzschild M Ascherio A. Serum cholesterol and the progression of Parkinson's disease: results from DATATOP. PLoS ONE. (2011) 6:e22854. 10.1371/journal.pone.0022854 21853051 PMC3154909

[22] Marek K Jennings D Lasch S Siderowf A Tanner C Simuni T. The Parkinson's Progression Markers Initiative (PPMI). Prog Neurobiol. (2011) 95:629–35. 10.1016/j.pneurobio.2011.09.005 21930184 PMC9014725

[23] Malek N Swallow D Grosset KA Lawton MA Marrinan SL Lehn AC. Tracking Parkinson's: study design and baseline patient data. J Parkinson's Dis. (2015) 5:947–59. 10.3233/JPD-150662 26485428 PMC4927877

[24] Stephenson D Hu MT Romero K Breen K Burn D Ben-Shlomo Y. Precompetitive data sharing as a catalyst to address unmet needs in Parkinson's disease 1. J. Parkinson's Dis. (2015) 5:581–94. 10.3233/JPD-150570 26406139 PMC4887129

[25] Goetz CG Tilley BC Shaftman SR Stebbins GT Fahn S Martinez-Martin P. Movement disorder society-sponsored revision of the unified Parkinson's disease rating scale (MDS-UPDRS): scale presentation and clinimetric testing results. Movement Disord. (2008) 23:2129–70. 10.1002/mds.22340 19025984

[26] Wilson PF. Cardiovascular disease risk assessment for primary prevention in adults: Our approach [Internet] UpToDate. (2020).

[27] D'agostino RB Vasan RS Pencina MJ Wolf PA Cobain M Massaro JM. General cardiovascular risk profile for use in primary care. Circulation. (2008) 117:743–53. 10.1161/CIRCULATIONAHA.107.699579 18212285

[28] Greenland S Pearl J Robins JM. Causal diagrams for epidemiologic research. Epidemiology. (1999) 10:37–48. 10.1097/00001648-199901000-00008 9888278

[29] Pearl J. Causality. Cambridge: Cambridge University Press (2009).

[30] Wills A-M Li R Pérez A Ren X Boyd J Investigators NN-P. Predictors of weight loss in early treated Parkinson's disease from the NET-PD LS-1 cohort. J Neurol. (2017) 264:1746–53. 10.1007/s00415-017-8562-4 28712000 PMC5789795

[31] Wills A-MA Pérez A Wang J Su X Morgan J Rajan SS. Association between change in body mass index, unified Parkinson's disease rating scale scores, and survival among persons with Parkinson disease: secondary analysis of longitudinal data from NINDS exploratory trials in Parkinson disease long-term study 1. JAMA Neurol. (2016) 73:321–8. 10.1001/jamaneurol.2015.4265 26751506 PMC5469290

[32] Cumming K Macleod AD Myint PK Counsell CE. Early weight loss in parkinsonism predicts poor outcomes: evidence from an incident cohort study. Neurology. (2017) 89:2254–61. 10.1212/WNL.0000000000004691 29079685 PMC5705250

[33] Uc EY Struck LK Rodnitzky RL Zimmerman B Dobson J Evans WJ. Predictors of weight loss in Parkinson's disease. Movement Disord. (2006) 21:930–6. 10.1002/mds.20837 16534756

[34] Shulman LM Gruber-Baldini AL Anderson KE Fishman PS Reich SG Weiner WJ. The clinically important difference on the unified Parkinson's disease rating scale. Arch Neurol. (2010) 67:64–70. 10.1001/archneurol.2009.295 20065131

[35] Curioni C Lourenco P. Long-term weight loss after diet and exercise: a systematic review. Int J Obes. (2005) 29:1168–74. 10.1038/sj.ijo.0803015 15925949

[36] van der Kolk NM de Vries NM Kessels RP Joosten H Zwinderman AH Post B. Effectiveness of home-based and remotely supervised aerobic exercise in Parkinson's disease: a double-blind, randomised controlled trial. Lancet Neurol. (2019) 18:998–1008. 10.1016/S1474-4422(19)30285-6 31521532

[37] Hernán MA Taubman SL. Does obesity shorten life? The importance of well-defined interventions to answer causal questions. Int J Obesity. (2008) 32:S8–14. 10.1038/ijo.2008.82 18695657

[38] Qiu C Winblad B Fratiglioni L. The age-dependent relation of blood pressure to cognitive function and dementia. Lancet Neurol. (2005) 4:487–99. 10.1016/S1474-4422(05)70141-1 16033691

[39] Stewart R Xue Q-L Masaki K Petrovitch H Ross GW White LR. Change i blood pressure and incident dementia: a 32-year prospective study. Hypertension. (2009) 54:233–40. 10.1161/HYPERTENSIONAHA.109.128744 19564551 PMC3136040

[40] van den Heuvel L Evers LJ Meinders MJ Post B Stiggelbout AM Heskes TM. Estimating the effect of early treatment initiation in Parkinson's disease using observational data. Movement Disord. (2021) 36:407–14. 10.1002/mds.28339 33107639 PMC7984449

[41] Mahmood SS Levy D Vasan RS Wang TJ. The Framingham Heart Study and the of cardiovascular disease: a historical perspective. Lancet. (2014) 383:999–1008. 10.1016/S0140-6736(13)61752-3 24084292 PMC4159698

[42] Baumgart M Snyder HM Carrillo MC Fazio S Kim H Johns H. Summary of the evidence on modifiable risk factors for cognitive decline and dementia: a population-based perspective. Alzheimer's Dementia. (2015) 11:718–26. 10.1016/j.jalz.2015.05.016 26045020

