# Supplementary material for: The effect of cardiovascular risk on disease progression in de novo Parkinson's disease patients: An observational analysis
Source: Front Neurol. 2023 Apr 12;14:1138546. doi: 10.3389/fneur.2023.1138546 (PMC10130532; doi:10.3389/fneur.2023.1138546)
Supplement: Supplementary file 1 [file Data_Sheet_1.DOCX]

# Supplement

**Table 1s:** cross sectional studies on cardiovascular risk and Parkinson’s disease progression. MoCA = Montreal Cognitive Assessment, H&Y = Hoehn & Yahr, LEDD = levodopa equivalent daily dose, PD = Parkinson’s disease, cvd = cardiovascular disease, DTBZ = dihydrotetrabenazine, HVLT = Hopkins Verbal Learning Test.

| **Study** | **Population** | **Outcome measures** | **Exposures** | **Model** | **Results** |
| --- | --- | --- | --- | --- | --- |
| Jones et al. (2012) (1) | 1948 PD subjects from NPF-QII registry | Semantic fluency and a 5-word recall  memory task. | Heart / circulation problems, diabetes, arthritis, cancer,  respiratory disease, and other neurologic disease, clinician rated for presence and severity. | Multiple hierarchical regression controlling for demographic, disease duration, severity (H&Y) and medication status | Severity of heart / circulation problems independently contributed to worse delayed recall performance (β = -0.05, p = 0.03) |
| Jones et al. (2014) (2) | 314 PD subjects recruited via MD center in Florida | Digit span, HVLT-R, Boston Naming Test, TMT-A/B, Stroop  Color-Word Test, COWA, Stroop Single Word Reading. | Systolic blood pressure, hypertension, cardio-  vascular history | Multiple hierarchical regression controlling for UPDRS motor scores, disease duration and LED. Sensitivity analysis also controls for age and education. | Health comorbidities in general was not significant for any cognitive domain. (Controlled) hypertension was associated with worse executive function and delayed verbal memory scores.  Higher pulse pressure values significantly related to worse executive function (β = -0.133, p =0.043), delayed verbal memory (β = -0.138, p = 0.010) and processing speed scores (β = -0.128, p = 0.017) as UPDRS motor scores increased in  severity. |
| Jones et al. (2017) (3) | 67 PD, 61 non-PD subjects recruited via MD center in Florida | Z-scores of Trail Making Test - Part A and B, Animal Fluency, Boston Naming Test, 30-min delayed recall, HVLT-II, Forward Span and Backward Span | Framing risk score (FRS), leukoaraiosis volume. | Hierarchical regressions including covariates on group,  exposure and interaction depending on the research question. | Higher FCR correlated with worse executive functioning, in both PD and non-PD. Leukoaraiosis correlated with both FCR and executive functioning in non-PD, but not in PD. |
| Swallow et al. (2016) (4) | 2909 PD sub-  jects at BL from  Tracking Parkin-  son’s and Ox-  ford Parkinson’s  Disease Centre  (OPDC) cohorts | MoCA, UPDRS part III | QRISK2 (high, medium, low) | Generalized linear regression  controlling for age, sex, disease duration and coffee use | Increasing vascular risk and CVD were associated with worse motor score (p<0.001) and more cognitive impairment (p<0.001) |
| Malek et al. (2016) (5) | 1759 PD subjects  at BL from Tracking Parkinson’s | MoCA,  UPDRS part III | QRISK2, history of cvd, hypertension, cholesterol, obesity, diabetes, smoking, leukoaraiosis, lacunar and territorial infarctions. | Generalized linear regression adjusted for LEDD/drug  naiveté, age, gender, disease duration. | More than 2 vascular risk factors associated with worse MDS-UPDRS 3 motor scores (β = 4.05, p  = 0.003) and with cognitive impairment (ordinal odds ratio 2.24, p = 0.002). QRISK2 > 20 associated with motor severity (β = 3.41, p ¡ 0.001). White matter leukoaraiosis (but not lacunar or  territorial infarction) associated with impaired cognition (p = 0.006). |
| Kotagal et  al. (2014) (6) | 85 PD subjects  from University of  Michigan Medical  Center and the  Veterans Affairs  Ann Arbor | MDS-UPDRS part III (off state), leukoaraiosis, nigrostriatal  dopamine terminal loss. | Framingham risk score (FRS) | Multivariable linear regression adjusted for sex, MoCA,  striatal DTBZ and frontal leukoaraiosis severity. | Elevated FRS was not associated with MDS-UPDRS part III score after controlling for confounders. Frontal leukoaraiosis was associated with the rate of axial and total MDS-UPDRS scores per year of symptoms |
| Kotagal et  al. (2014) (7) | 83 PD, 49 non-PD  subjects from University of Michigan | Timed-Up and Go | Framingham risk score (FRS) | Multivariable linear regression adjusted for striatal DTBZ  and frontal leukoaraiosis  severity. | Elevated FRS associated with slower Timed Up and Go test performance (compared to normal range risk scores). This was not observed in healthy controls. |
| Papa-  petropou-  los et al.  (2004) (8) | 167 late onset PD  subjects from Department of Neurology of Patras  University | H&Y score | Minor stroke, ischemic heart disease, diabetes. | Logistic analyses adjusted for  age, sex, disease duration, the  presence of depression and  dementia. | H&Y score was significantly higher in PD subjects who suffered from minor stroke, ischemic heart disease or diabetes compared to subjects  without such comorbidity. |

**Table 2s:** longitudinal studies on cardiovascular risk and Parkinson’s disease progression. MoCA = Montreal Cognitive Assessment, H&Y = Hoehn & Yahr, LEDD = levodopa equivalent daily dose, LNS = Letter Number Sequencing, PD = Parkinson’s disease, BMI = body mass index, BL = baseline, MMSE = Mini-Mental State Examination, CAD = coronary artery disease, SDMT = Symbol Digit Modalities Test, HVLT = Hopkins Verbal Learning Test, BJLO = Benton Judgment of Line Orientation, PDD = Parkinson’s disease dementia

| **Study** | **Population** | **Outcome measures** | **Exposures** | **Model** | **Results** |
| --- | --- | --- | --- | --- | --- |
| Sterling et al. (2015) (9) | 64 PD, 64 HC sub-  jects measured at  BL, 18, 36 months | Z-scores of Grooved Pegboard Test, BVMT-R, HVLT-R, Design Fluency Test, Verbal Fluency Test, CWInt-Switch  and CWInt-Inhibition sub-  tests, Digit Span, Spatial Span, LNS, CWInt color symbol search, Boston Naming Test, CWInt-Word sub-test, BJLO | LDL cholesterol at BL | Linear mixed model with cognitive  score as dependent variable and random slope and intercept for years  past. Correcting for age, LEDD,  group, education years, depression  score, statin usage and cholesterol  level. Interactions terms: statin usage  × cholesterol level, group × year,  year × cholesterol level, group ×  cholesterol level, years × group ×  cholesterol level | Higher LDL-cholesterol levels associated with improved executive set shifting (β = 0.003, p = 0.001) and  fine motor scores (β = 0.002, p = 0.030) over time. This improved executive set shifting was PD specific. |
| Huang et al. (2015) (10) | 774 PD subjects  from DATATOP  study | Time till  dopaminergic therapy. | Serum cholesterol levels at BL | Hazard ratios correcting for gender, treatment group (deprenyl or not), age, uric acid concentration, PD sub type (tremor, PIGD or mixed), BMI. | HR for one standard deviation (SD) increase in serum cholesterol = 0.90  [(0.80–1.01), p = 0.09. Only found in males (HR = 0.88), not in females (HR = 1.03) |
| Yoo et al. (2019) (11) | 70 PD subjects (de novo)  followed for 6 years. From Movement Disorders  outpatient clinic  in the Yonsei Uni-  versity Severance  Hospital | CDR-SOB, MMSE, UPDRS part II  and III, time to PDD | BMI at BL | Linear mixed model and hazard ratios adjusted for age at PD onset, sex, years of education, UPDRS part III, clinical phenotype. | Overweight and obese groups showed slower progression of cognitive decline in global cognitive function as well  as language and memory domains. Overweight and obese group showed  a lower risk of developing dementia compared with the under-/normal-weight group (HR = 0.36, p = 0.046) |
| Kim et al. (2019) (12) | 399 PD subjects  from PPMI, data  up to 5 years | Swab and England score (primary),  MDS-UPDRS part III (off) and MoCA (secondary) | BMI at BL | Linear mixed model with time, BMI  interaction adjusted for age, sex, disease duration, history of diabetes and BL score dependent variable. | Rate of change greater in obesity (p < 0.001) and overweight group (p = 0.004) compared to normal/underweight group. Risk of functional dependency also higher in obese patients in HR analysis (HR = 2.63, p = 0.006). |
| Chahine et al. (2019) (13) | 141 PD, 63 HC  subjects from  PPMI cohort with  adequate MRI  scan. BL visit,  one follow up (24  months) | MoCA, HVLT delayed/recognition,  BJLO, LNS, semantic fluency, SDMT, number of white matter intensities. | Modified Framingham risk score (mFRS), white matter intensities (all at BL) | Linear mixed model adjusted for age,  sex, cognitive test score at BL, disease duration at BL, years of education with random intercept for visit. | Higher mFRS and white matter hyperintensities associated with greater annual rate of change in MoCA (β =-0.040, p=0.007 and (β = -0.029, p =  0.049). |
| Doiron et al. (2017) (14) | 367 PD subjects  from PPMI cohort at BL and 24  months | Z-scores of MoCA, BJLO, HVLT delayed/recognition, LNS, semantic fluency, SDMT. | History/length of  hypertension, pulse pressure, diabetes, BMI (all at BL) | Generalized mixed models controlling  for age, education level, disease duration, motor symptoms, depressive symptoms, LEDD | Longer history hypertension and higher pulse pressure predict lower z-scores on immediate and delayed  free recall, recognition, and verbal fluency tests. |
| Mollenhauer et al. (2019) (15) | 135 PD (de novo),  109 HC from  DeNoPa study followed for 4 years | MDS-UPDRS part III (on), MMSE. | Diabetes, smoking, blood pressure, CAD, hypertension, smoking, BMI, (LDL/HDL) cholesterol levels, glucose levels, diabetes,  alcohol abuse (all at BL) | Latent growth curve model, did not  correct for any confounders in main analysis. | Diabetes and hypertension were found to be predictors of faster cognitive decline, both in control and PD, whereas fasting glucose was PD specific. Orthostatic blood pressure drop, higher systolic blood pressure and coronary artery disease were associated with faster motor progression |

## Figure 1s: DAGs

Directed acyclic graph (DAG) reflecting the assumed relationships between variables for the analysis of the effect of each exposure on PD progression. Relationships between confounders are not shown to avoid clutter (and because these did not alter the required adjustment sets). The DAGs were constructed together with multiple clinical PD experts. DAGs for the other exposures are displayed in the supplement. SES: socioeconomic status, cv: cardiovascular.

##
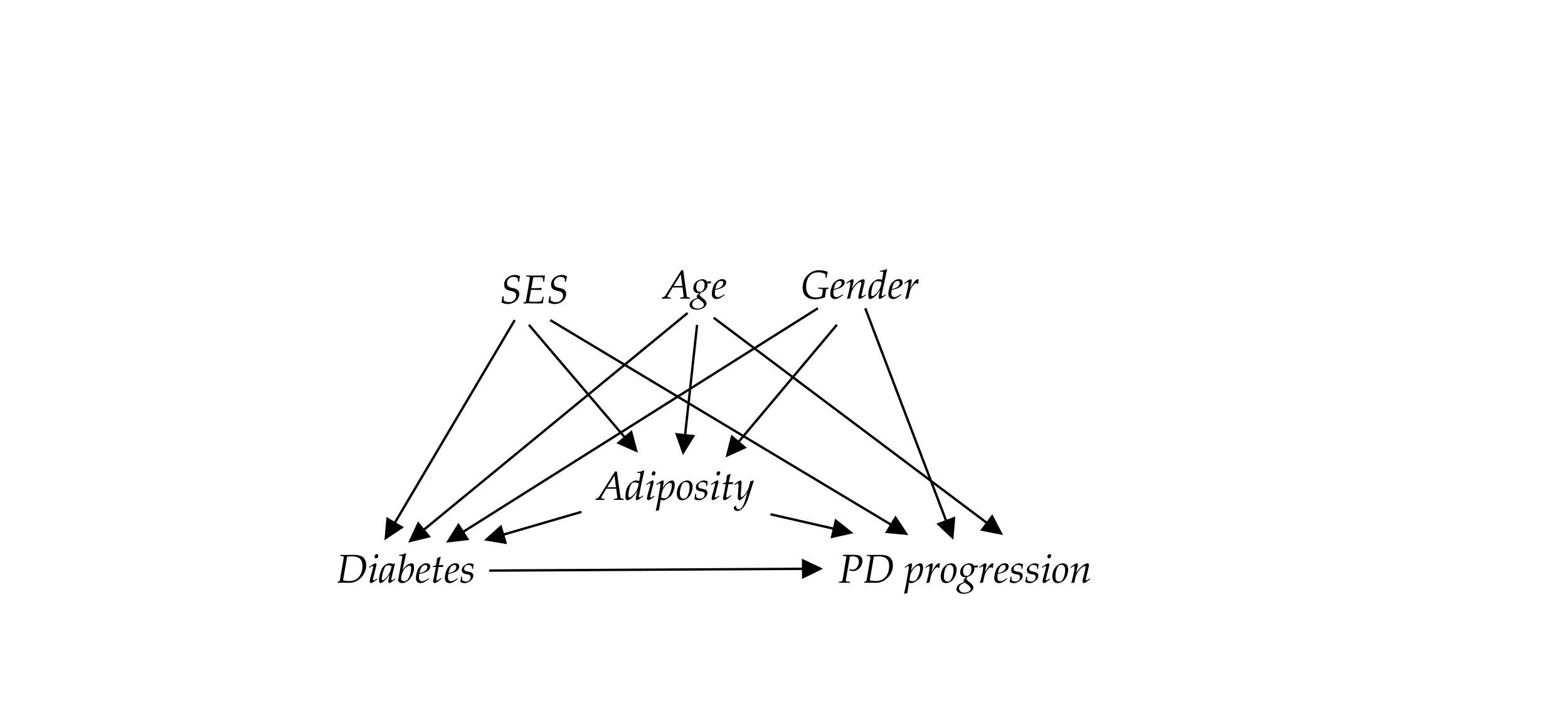

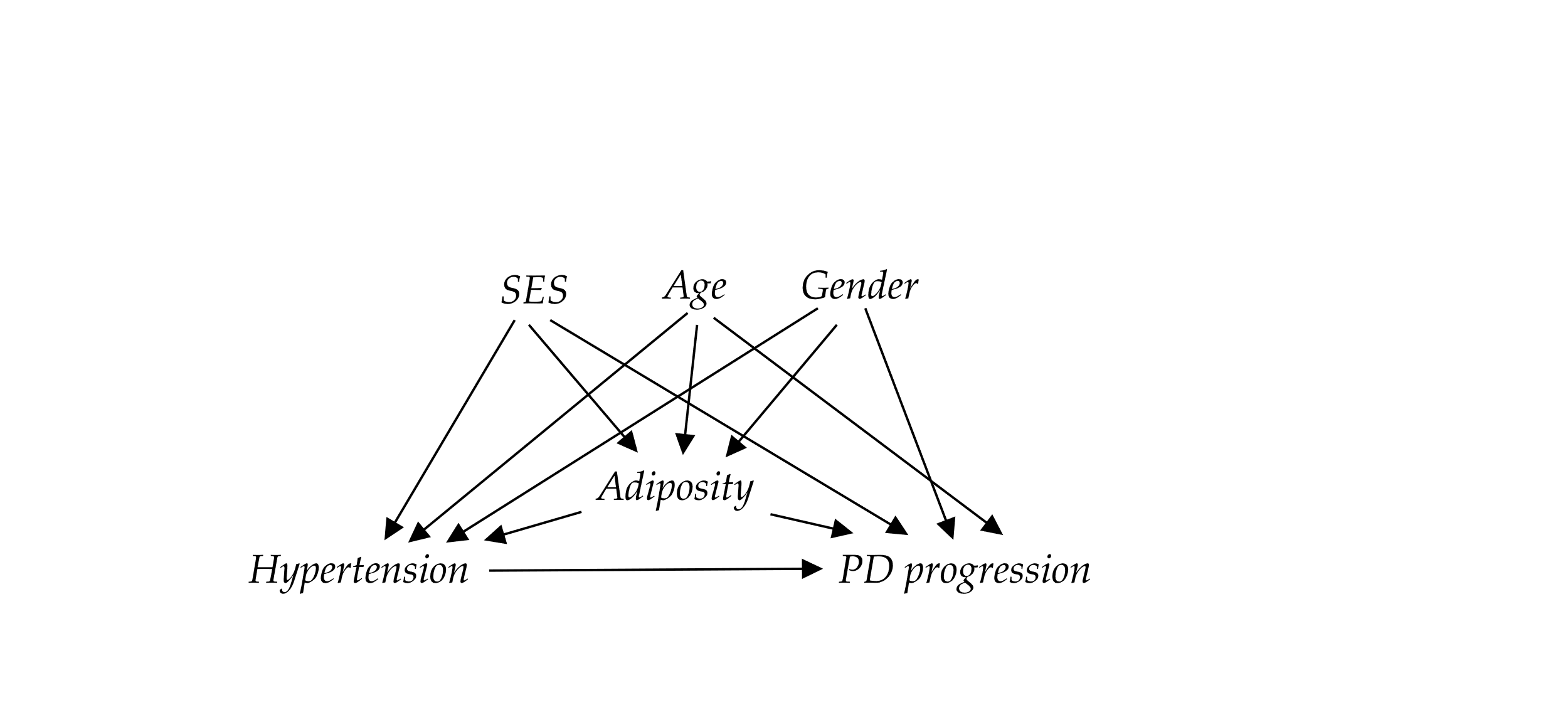

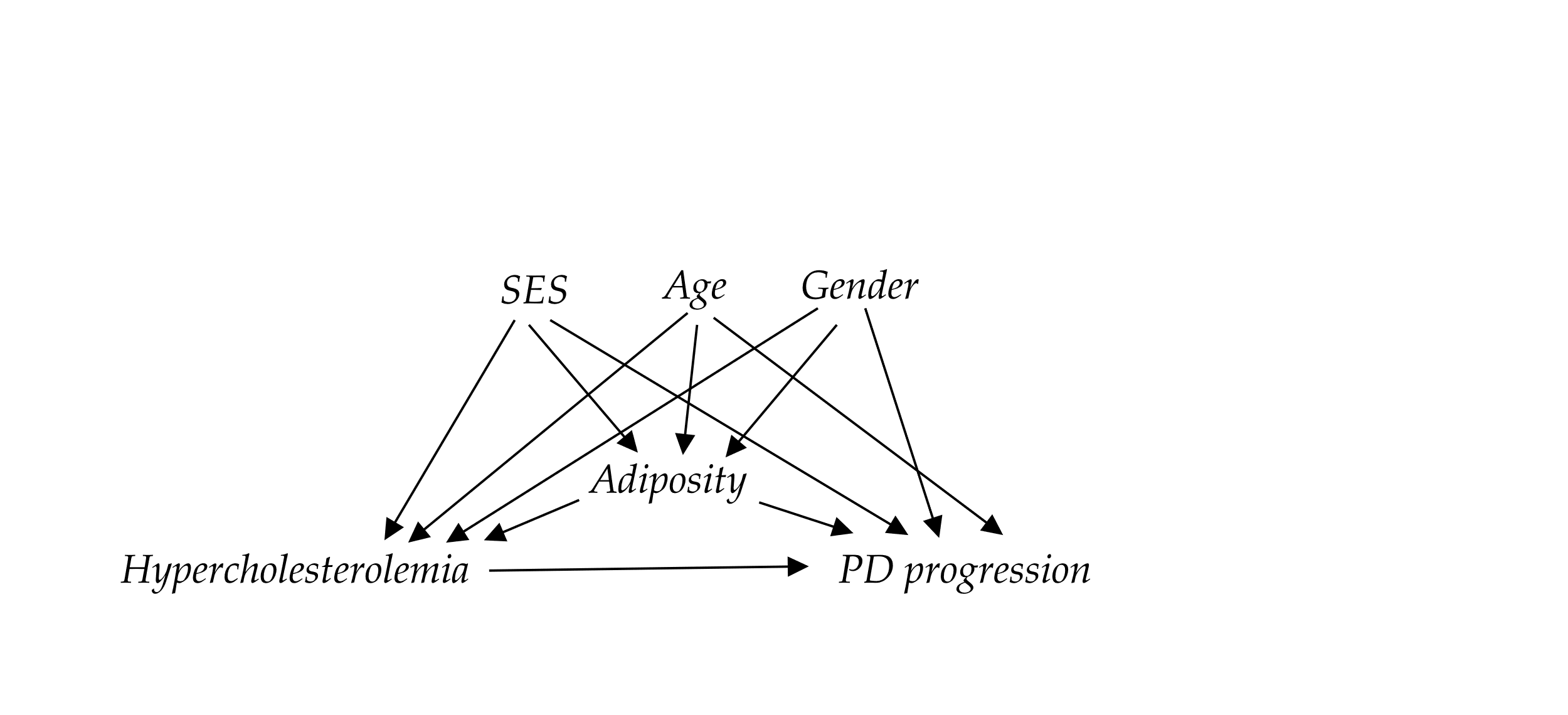

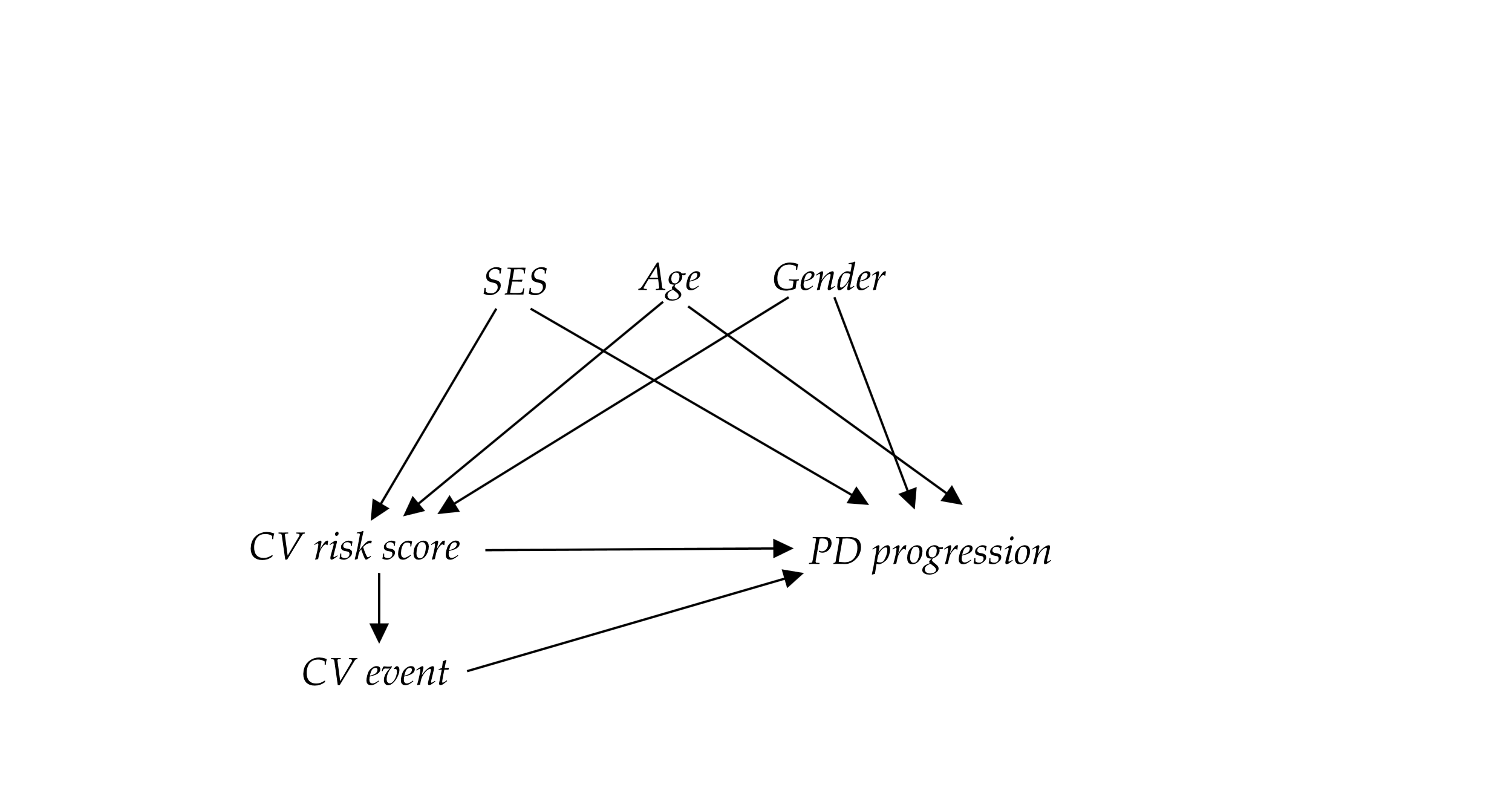


## Table 3s: full results PPMI

Estimated effects of each exposure on the rate of change of each outcome measure per year in the PPMI cohort. MDS-UPDRS: Movement Disorders Society Unified Parkinson Disease Rating Scale, MoCA: Montreal Cognitive Assessment, SDMT: Symbol Digit Modalities Test, LNS: Letter-Number Sequencing task, mFrs: modified Framingham risk score, BMI: body mass index, cveh: cardiovascular event history, HC: healthy control, PD: Parkinson’s disease, CI = confidence interval.

| MDS-UPDRS PART III - PD | unadjusted | | | adjusted | | |
| --- | --- | --- | --- | --- | --- | --- |
|  | **beta** | **p** | **95% CI** | **beta** | **p** | **95% CI** |
| Year:BMI | 0.06615 | 0.002 | 0.02331 — 0.109 | 0.05948 | 0.006 | 0.01719 — 0.1018 |
| Year:cveh | 0.4559 | 0.324 | -0.4506 — 1.363 | 0.3608 | 0.428 | -0.5302 — 1.252 |
| Year:diabetes | 0.6364 | 0.167 | -0.2648 — 1.538 | 0.4444 | 0.316 | -0.4223 — 1.311 |
| Year:hypercholesterolemia | 0.2627 | 0.204 | -0.1427 — 0.668 | 0.08167 | 0.685 | -0.3122 — 0.4755 |
| Year:hypertension | 0.134 | 0.518 | -0.2718 — 0.5397 | -0.0601 | 0.776 | -0.4737 — 0.3535 |
| Year:mFRS | 0.03212 | 0.137 | -0.01024 — 0.07449 | 0.01204 | 0.733 | -0.05702 — 0.08111 |

| MDS-UPDRS PART III - HC | unadjusted | | | adjusted | | |
| --- | --- | --- | --- | --- | --- | --- |
|  | **beta** | **p** | **95% CI** | **beta** | **p** | **95% CI** |
| Year:BMI | 0.01067 | 0.349 | -0.01162 — 0.03297 | 0.009745 | 0.362 | -0.01103 — 0.03052 |
| Year:cveh | -0.03489 | 0.910 | -0.6424 — 0.5726 | -0.2191 | 0.467 | -0.8075 — 0.3693 |
| Year:diabetes | 0.3083 | 0.188 | -0.1502 — 0.7668 | 0.2844 | 0.218 | -0.1661 — 0.7348 |
| Year:hypercholesterolemia | -0.02996 | 0.771 | -0.2315 — 0.1716 | -0.1276 | 0.206 | -0.3242 — 0.06907 |
| Year:hypertension | 0.1266 | 0.211 | -0.0715 — 0.3247 | 0.01434 | 0.890 | -0.1887 — 0.2173 |
| Year:mFRS | 0.03179 | 0.001 | 0.01313 — 0.05045 | 0.001984 | 0.905 | -0.03048 — 0.03445 |

| MoCA - PD | unadjusted | | | adjusted | | |
| --- | --- | --- | --- | --- | --- | --- |
|  | **beta** | **p** | **95% CI** | **beta** | **p** | **95% CI** |
| Year:BMI | -0.001504 | 0.805 | -0.01342 — 0.01041 | -0.002757 | 0.658 | -0.01492 — 0.009406 |
| Year:cveh | -0.07948 | 0.537 | -0.332 — 0.173 | 0.04929 | 0.707 | -0.2068 — 0.3054 |
| Year:diabetes | -0.2766 | 0.029 | -0.5246 — -0.02859 | -0.1808 | 0.159 | -0.4316 — 0.07003 |
| Year:hypercholesterolemia | -0.02852 | 0.611 | -0.1382 — 0.08119 | 0.0321 | 0.575 | -0.07993 — 0.1441 |
| Year:hypertension | -0.1333 | 0.017 | -0.2423 — -0.02438 | -0.04981 | 0.402 | -0.1662 — 0.06655 |
| Year:mFRS | -0.02518 | <0.001 | -0.03643 — -0.01394 | 0.001671 | 0.866 | -0.01777 — 0.02111 |

| MoCA - HC | unadjusted | | | adjusted | | |
| --- | --- | --- | --- | --- | --- | --- |
|  | **beta** | **p** | **95% CI** | **beta** | **p** | **95% CI** |
| Year:BMI | -0.008727 | 0.076 | -0.01834 — 0.0008907 | -0.008908 | 0.075 | -0.01864 — 0.0008234 |
| Year:cveh | -0.1121 | 0.423 | -0.386 — 0.1619 | -0.05109 | 0.716 | -0.3249 — 0.2228 |
| Year:diabetes | -0.158 | 0.128 | -0.3612 — 0.04532 | -0.09765 | 0.359 | -0.3054 — 0.1102 |
| Year:hypercholesterolemia | 0.01269 | 0.772 | -0.07289 — 0.09826 | 0.04393 | 0.324 | -0.04295 — 0.1308 |
| Year:hypertension | -0.05411 | 0.207 | -0.1381 — 0.02985 | 0.01929 | 0.676 | -0.07065 — 0.1092 |
| Year:mFRS | -0.01339 | <0.001 | -0.02089 — -0.005895 | 0.0002191 | 0.976 | -0.0139 — 0.01433 |

| SDMT - PD | unadjusted | | | adjusted | | |
| --- | --- | --- | --- | --- | --- | --- |
|  | **beta** | **p** | **95% CI** | **beta** | **p** | **95% CI** |
| Year:BMI | -0.02627 | 0.113 | -0.05874 — 0.006202 | -0.02359 | 0.182 | -0.05817 — 0.01098 |
| Year:cveh | 0.02499 | 0.944 | -0.6708 — 0.7208 | 0.4122 | 0.242 | -0.2774 — 1.102 |
| Year:diabetes | -0.6473 | 0.062 | -1.328 — 0.03288 | -0.2789 | 0.415 | -0.9479 — 0.39 |
| Year:hypercholesterolemia | -0.03856 | 0.801 | -0.339 — 0.2618 | 0.1621 | 0.289 | -0.1369 — 0.4611 |
| Year:hypertension | -0.2175 | 0.155 | -0.5172 — 0.0821 | 0.09073 | 0.571 | -0.2221 — 0.4036 |
| Year:mFRS | -0.07205 | <0.001 | -0.1028 — -0.04134 | -0.004829 | 0.857 | -0.0572 — 0.04754 |

| SDMT - HC | unadjusted | | | adjusted | | |
| --- | --- | --- | --- | --- | --- | --- |
|  | **beta** | **p** | **95% CI** | **beta** | **p** | **95% CI** |
| Year:BMI | 0.002878 | 0.865 | -0.03017 — 0.03592 | -0.0009009 | 0.961 | -0.03697 — 0.03517 |
| Year:cveh | -0.2793 | 0.558 | -1.212 — 0.6536 | -0.2578 | 0.613 | -1.254 — 0.738 |
| Year:diabetes | -0.1245 | 0.728 | -0.8249 — 0.576 | -0.3686 | 0.351 | -1.139 — 0.4024 |
| Year:hypercholesterolemia | 0.00231 | 0.988 | -0.2884 — 0.2931 | 0.08605 | 0.601 | -0.2352 — 0.4073 |
| Year:hypertension | 0.0312 | 0.831 | -0.2553 — 0.3177 | 0.01484 | 0.931 | -0.3206 — 0.3503 |
| Year:mFRS | -0.02129 | 0.107 | -0.04719 — 0.004599 | 0.002379 | 0.930 | -0.05031 — 0.05507 |

| LNS - PD | unadjusted | | | adjusted | | |
| --- | --- | --- | --- | --- | --- | --- |
|  | **beta** | **p** | **95% CI** | **beta** | **p** | **95% CI** |
| Year:BMI | -0.003863 | 0.329 | -0.01162 — 0.003892 | -0.002452 | 0.546 | -0.01039 — 0.00549 |
| Year:cveh | 0.03586 | 0.676 | -0.1323 — 0.2041 | 0.1242 | 0.147 | -0.04338 — 0.2918 |
| Year:diabetes | -0.05815 | 0.483 | -0.2207 — 0.1043 | -0.05162 | 0.534 | -0.2141 — 0.1109 |
| Year:hypercholesterolemia | -0.03047 | 0.405 | -0.1021 — 0.04119 | 0.02384 | 0.518 | -0.04824 — 0.09593 |
| Year:hypertension | -0.06507 | 0.074 | -0.1363 — 0.006189 | -0.01706 | 0.657 | -0.0923 — 0.05819 |
| Year:mFRS | -0.01549 | <0.001 | -0.02287 — -0.008111 | -0.002405 | 0.709 | -0.015 — 0.01019 |

| LNS - HC | unadjusted | | | adjusted | | |
| --- | --- | --- | --- | --- | --- | --- |
|  | **beta** | **p** | **95% CI** | **beta** | **p** | **95% CI** |
| Year:BMI | -0.007723 | 0.128 | -0.01766 — 0.00221 | -0.01272 | 0.017 | -0.02308 — -0.002362 |
| Year:cveh | -0.1886 | 0.186 | -0.4677 — 0.09051 | -0.165 | 0.262 | -0.452 — 0.1219 |
| Year:diabetes | -0.123 | 0.254 | -0.3341 — 0.08816 | -0.1087 | 0.336 | -0.329 — 0.1117 |
| Year:hypercholesterolemia | -0.01993 | 0.656 | -0.1076 — 0.06771 | -0.005587 | 0.906 | -0.09747 — 0.08629 |
| Year:hypertension | 0.06117 | 0.163 | -0.02468 — 0.147 | 0.08388 | 0.083 | -0.01033 — 0.1781 |
| Year:mFRS | -0.0001533 | 0.970 | -0.008186 — 0.007879 | 0.006111 | 0.430 | -0.008988 — 0.02121 |

## Table 4s: descriptive statistics baseline Tracking Parkinson’s cohort

Demographics, disease characteristics and cardiovascular risk factors at baseline of individuals included in the Tracking Parkinson’s analyses. CV = cardiovascular, PD = Parkinson’s disease, MoCA = Montreal Cognitive Assessment, MDS-UPDRS = Movement Disorders Society Unified Parkinson Disease Rating Scale.

|  | **PD (N=1841)** |
| --- | --- |
| **Age** |  |
| Mean (SD) | 67.4 (9.13) |
| Median [Min, Max] | 68.0 [31.0, 88.0] |
| **Sex** |  |
| Men | 1194 (64.9%) |
| **Education** |  |
| Less than 12 years | 594 (32.3%) |
| More than 12 years | 1247 (67.7%) |
| **Disease duration (years)** |  |
| Mean (SD) | 1.33 (0.904) |
| **CV event history** | 195 (10.6%) |
| **Hypertension** | 1061 (57.6%) |
| **Hypercholesterolemie** | 591 (32.1%) |
| **Antihypertensives** | 750 (40.7%) |
| **Modified Framingham risk score** |  |
| Mean (SD) | 15.6 (4.27) |
| **Diabetes** | 155 (8.4%) |
| **Systolic blood pressure (mmHg)** |  |
| Mean (SD) | 139 (19.0) |
| **Body mass index** |  |
| Mean (SD) | 27.0 (4.69) |
| **MDS-UPDRS part III** |  |
| Mean (SD) | 22.7 (12.1) |
| **MoCA** |  |
| Mean (SD) | 25.0 (3.47) |

**Figure 2s: observed progression on group level in Tracking Parkinson’s (median, 20%, and 40% around the median are shown; missing values were excluded).** **MDS-UPDRS: Movement Disorders Society Unified Parkinson Disease Rating Scale, MoCA: Montreal Cognitive Assessment.**
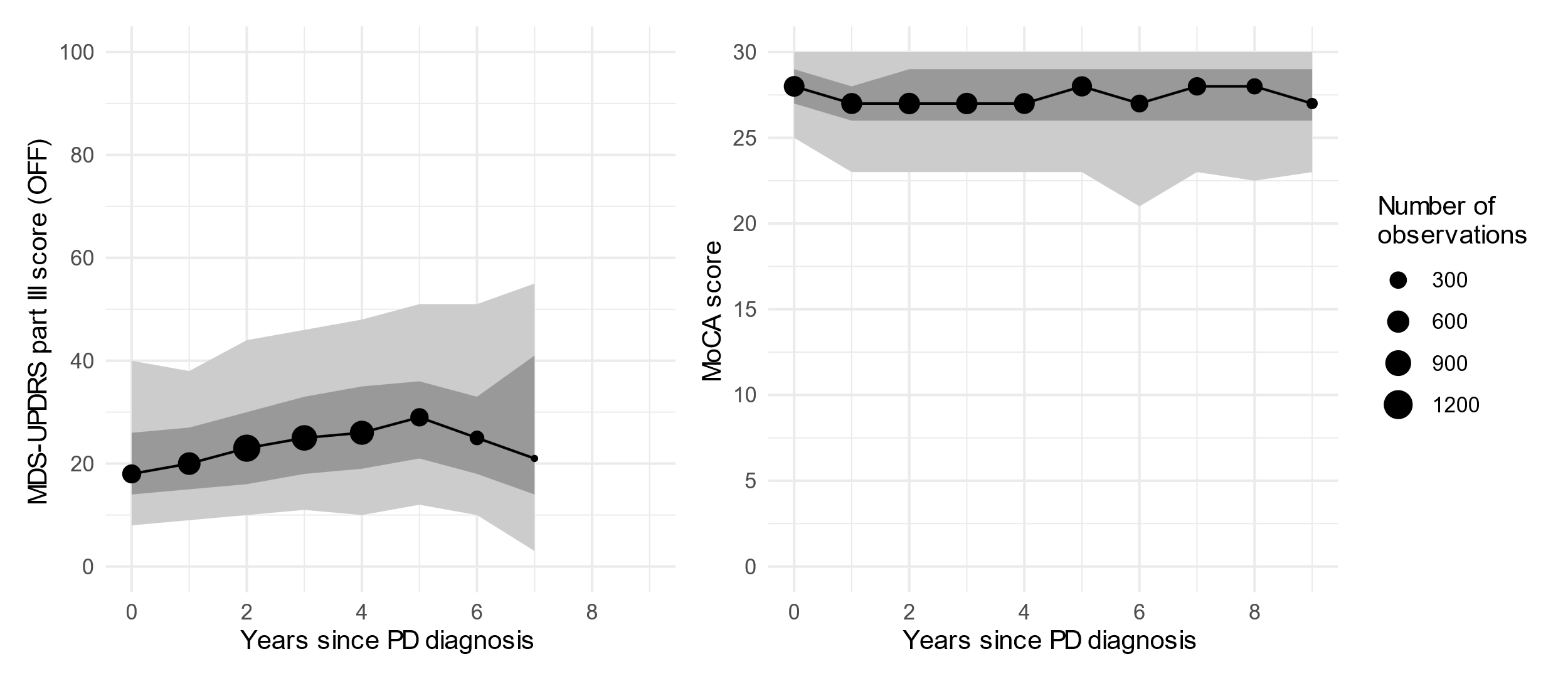


## Table 5s: full results Tracking Parkinson’s

Estimated effects of each exposure on the rate of change of each outcome measure per year in the Tracking Parkinson’s cohort. MDS-UPDRS: Movement Disorders Society Unified Parkinson Disease Rating Scale, MoCA: Montreal Cognitive Assessment, mFrs: modified Framingham risk score, BMI: body mass index, cveh: cardiovascular event history, HC: healthy control, PD: Parkinson’s disease, CI = confidence interval.

| MDS-UPDRS part III - PD | unadjusted | | | adjusted | | |
| --- | --- | --- | --- | --- | --- | --- |
|  | **beta** | **p** | **95% CI** | **beta** | **p** | **95% CI** |
| Year:BMI | 0.02615 | 0.325 | -0.02587 — 0.07817 | 0.01836 | 0.485 | -0.03306 — 0.06977 |
| Year:cveh | 0.5353 | 0.193 | -0.2712 — 1.342 | 0.6349 | 0.100 | -0.1201 — 1.39 |
| Year:diabetes | 0.2955 | 0.520 | -0.6036 — 1.195 | 0.239 | 0.575 | -0.5957 — 1.074 |
| Year:hypercholesterolemia | 0.3522 | 0.169 | -0.1499 — 0.8543 | 0.2871 | 0.246 | -0.1971 — 0.7712 |
| Year:hypertension | -0.266 | 0.273 | -0.7416 — 0.2095 | -0.4557 | 0.055 | -0.9205 — 0.008989 |
| Year:mFRS | 0.0307 | 0.290 | -0.02616 — 0.08756 | -0.05877 | 0.140 | -0.1368 — 0.01921 |

| MoCA - PD | unadjusted | | | adjusted | | |
| --- | --- | --- | --- | --- | --- | --- |
|  | **beta** | **p** | **95% CI** | **beta** | **p** | **95% CI** |
| Year:BMI | 0.004371 | 0.468 | -0.007425 — 0.01617 | 0.004789 | 0.428 | -0.007025 — 0.0166 |
| Year:cveh | -0.09836 | 0.304 | -0.2858 — 0.08913 | -0.08995 | 0.318 | -0.2663 — 0.08637 |
| Year:diabetes | -0.1271 | 0.235 | -0.337 — 0.08269 | -0.1134 | 0.267 | -0.3132 — 0.08641 |
| Year:hypercholesterolemia | -0.07833 | 0.196 | -0.1971 — 0.04043 | -0.08017 | 0.177 | -0.1963 — 0.03595 |
| Year:hypertension | -0.1657 | 0.003 | -0.2756 — -0.05591 | -0.08348 | 0.136 | -0.1931 — 0.02614 |
| Year:mFRS | -0.03709 | 0.000 | -0.05004 — -0.02414 | -0.009431 | 0.317 | -0.0279 — 0.009036 |

## Figure 3s: PPMI results vs Tracking Parkinson’s results, MDS-UPDRS part III

Estimated effect of each exposure on the rate of change in the MDS-UPDRS part III score per year. Dash dot line indicates threshold for assumed clinically relevant effect (see “Methods”). Intervals are 95% confidence intervals. mFrs: modified Framingham risk score, BMI: body mass index, cv: cardiovascular, HC: healthy control, PD: Parkinson’s disease.


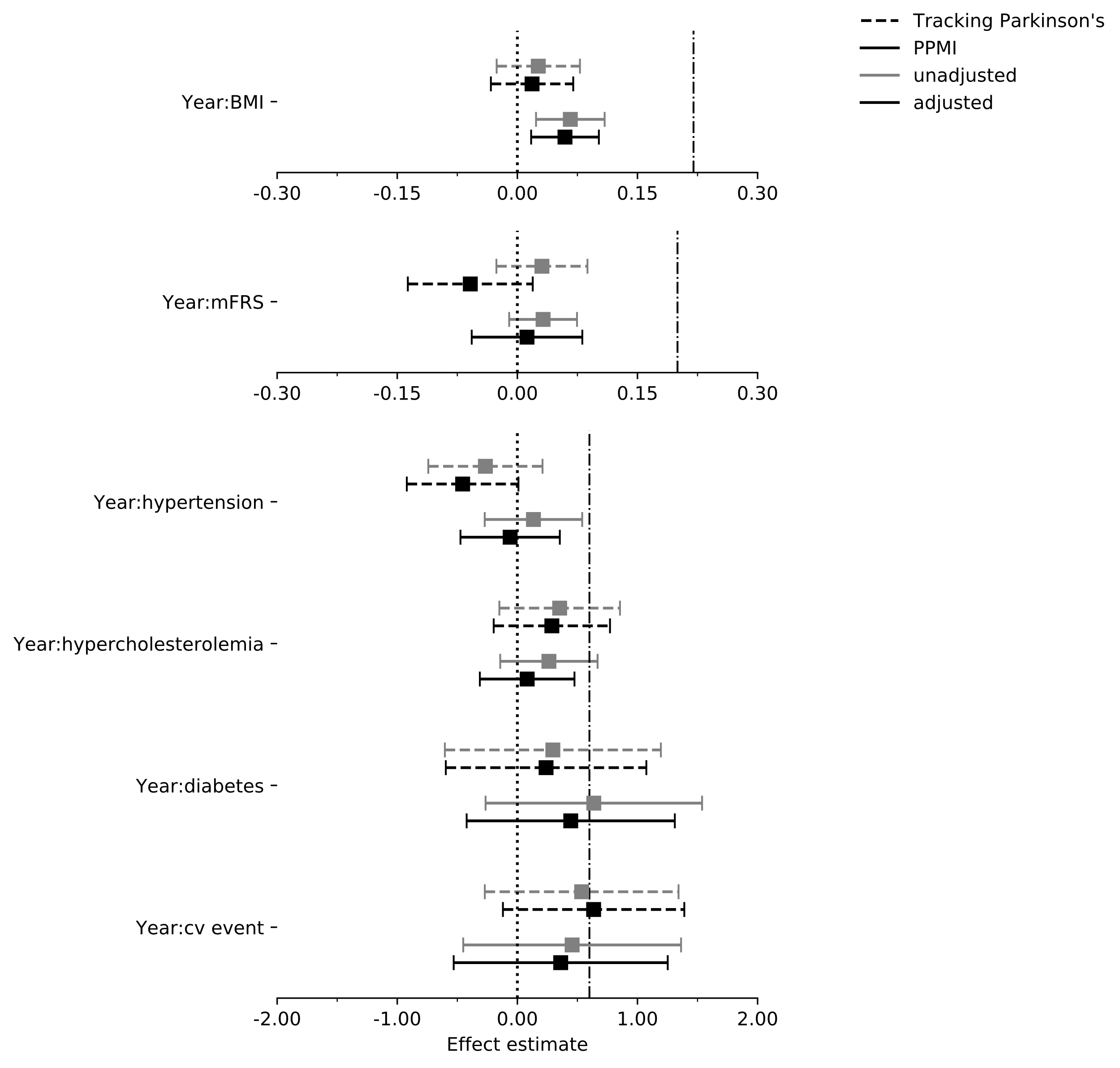


## Figure 4s: PPMI results vs Tracking Parkinson’s results, MoCA

Estimated effect of each exposure on the rate of change in the MoCA score per year. Intervals are 95% confidence intervals. mFrs: modified Framingham risk score, BMI: body mass index, cv: cardiovascular, HC: healthy control, PD: Parkinson’s disease.


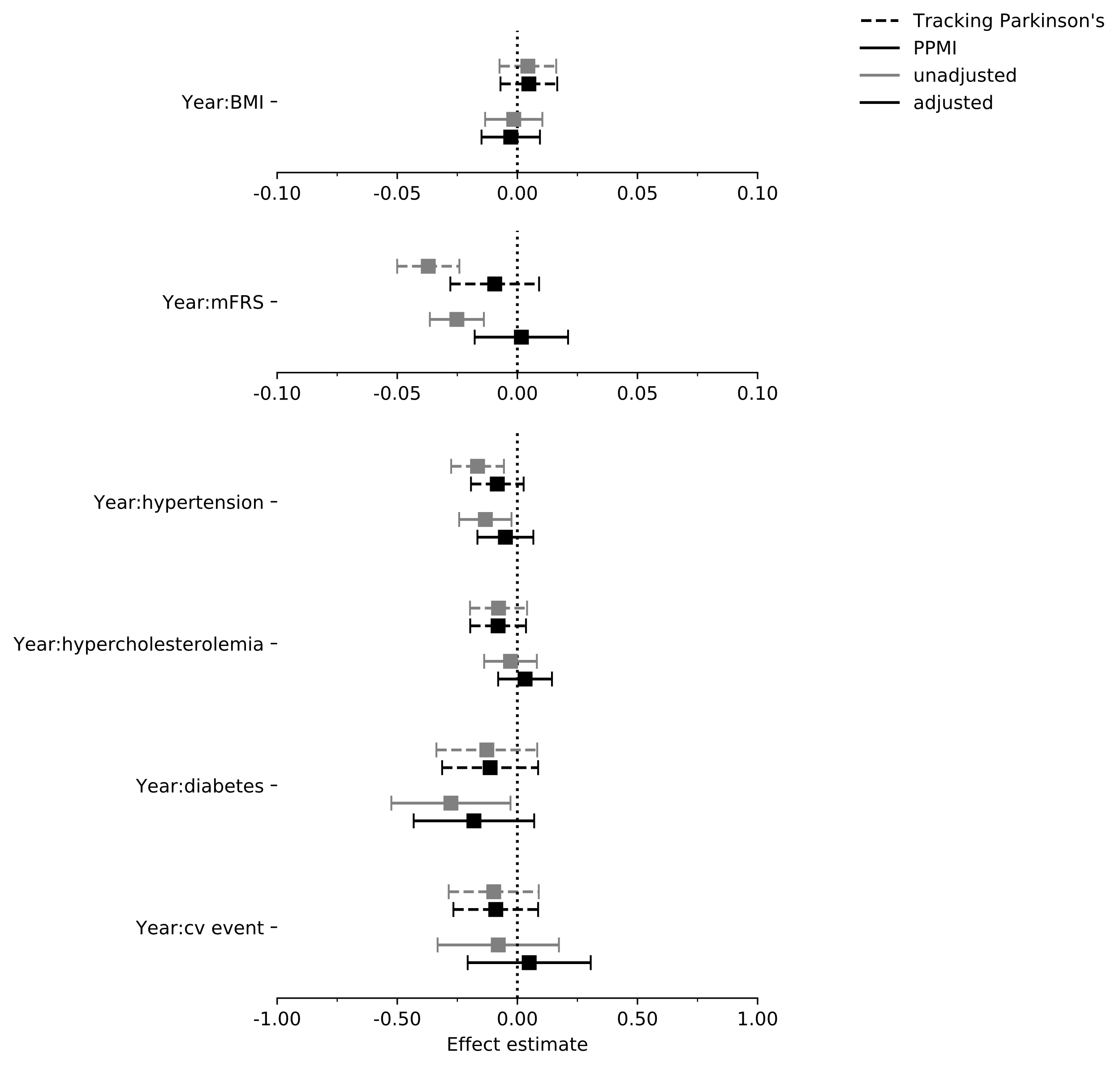


## Figure 5s: Examples of diagnostic plots for models based on the MDS-UPDRS part III data from PD subjects in the PPMI study.


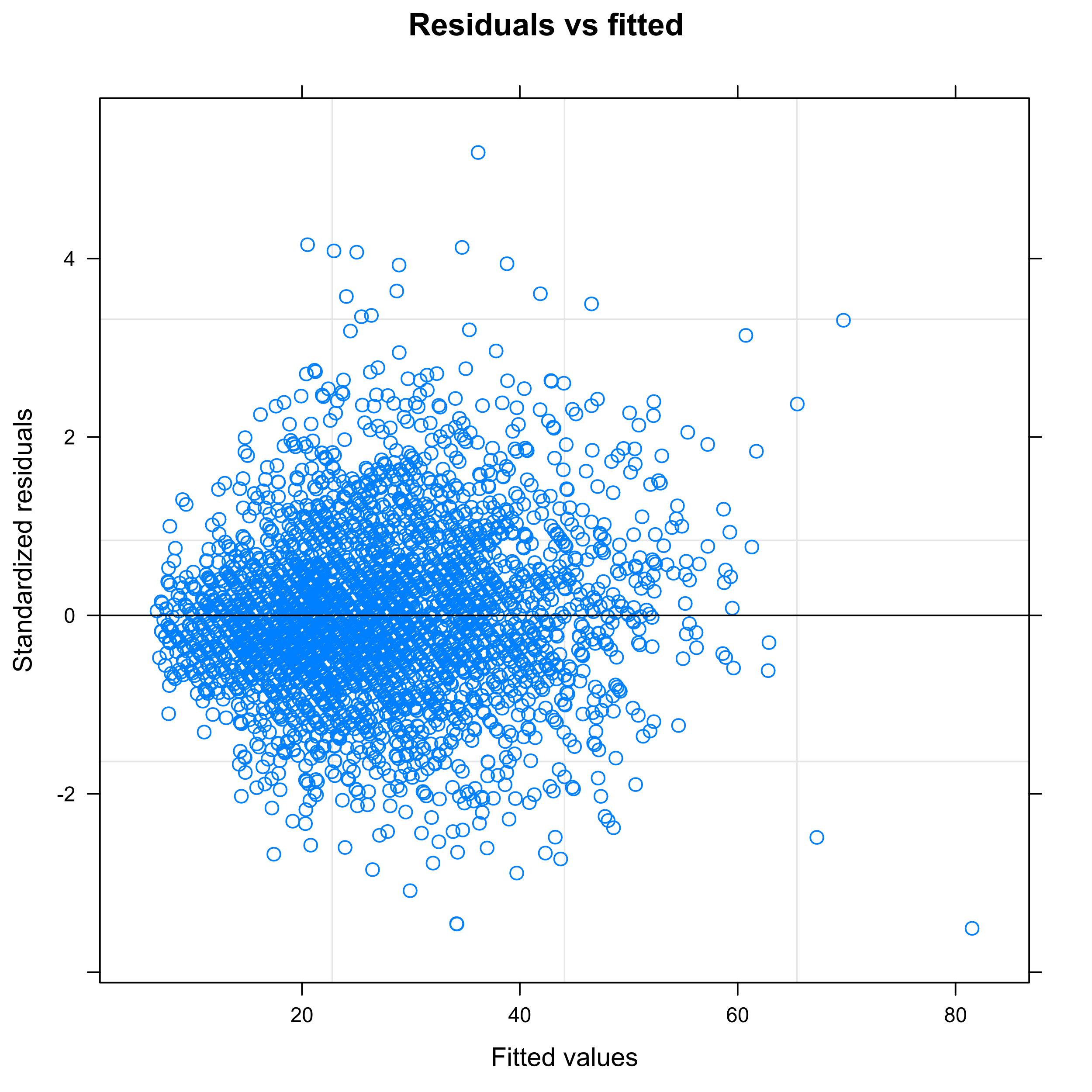

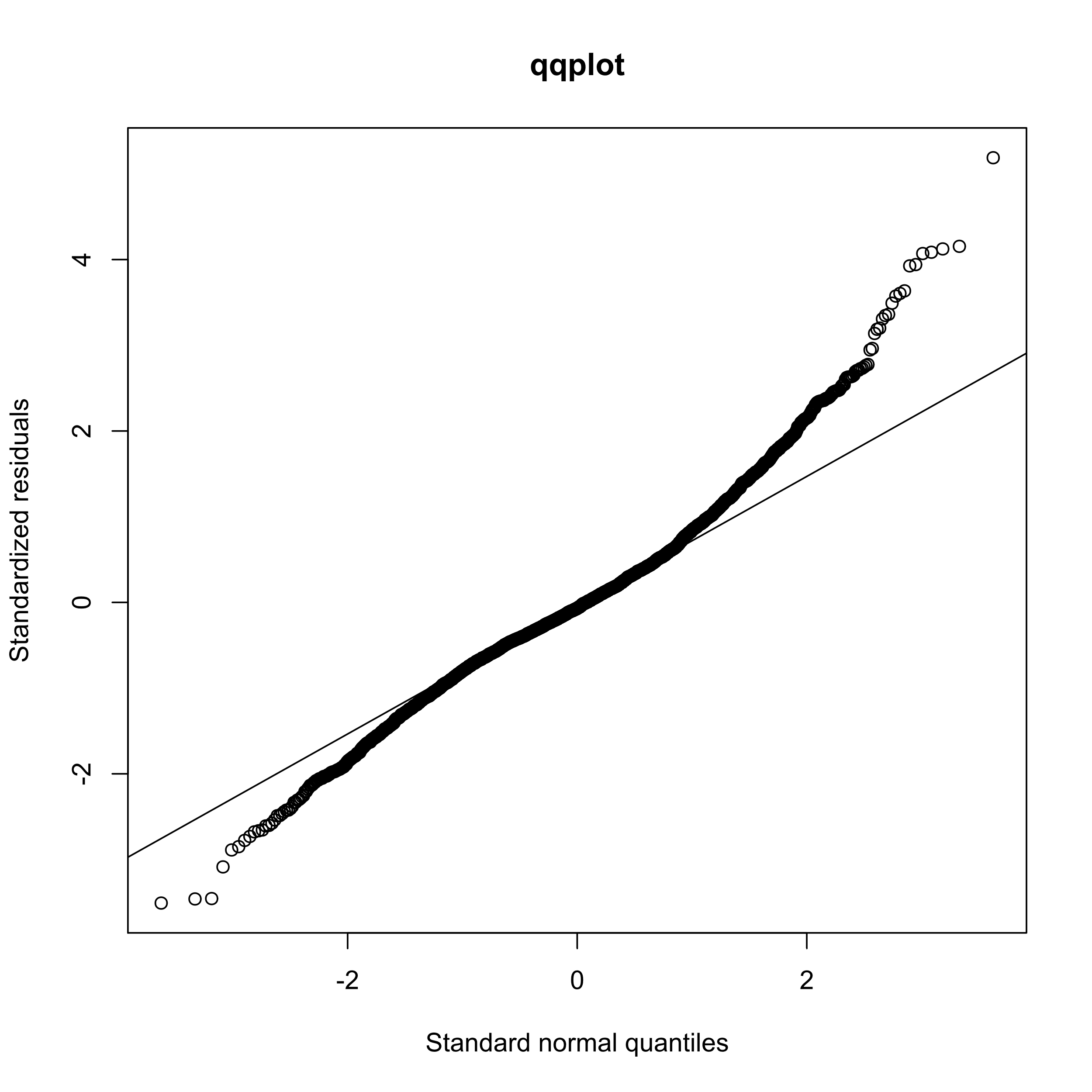

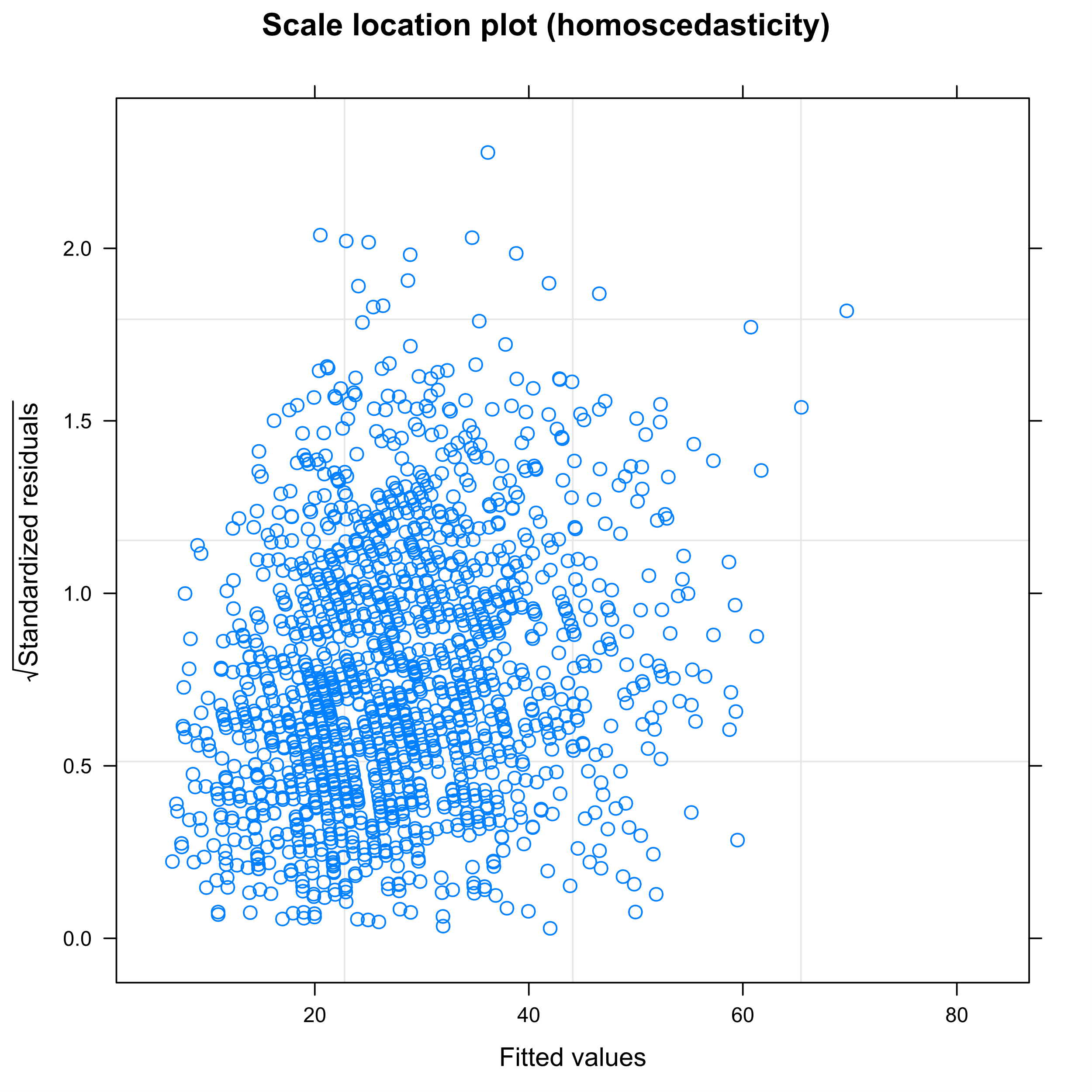


## Figure 6s: Examples of diagnostic plots for models based on the MoCA data from PD subjects in the PPMI study.


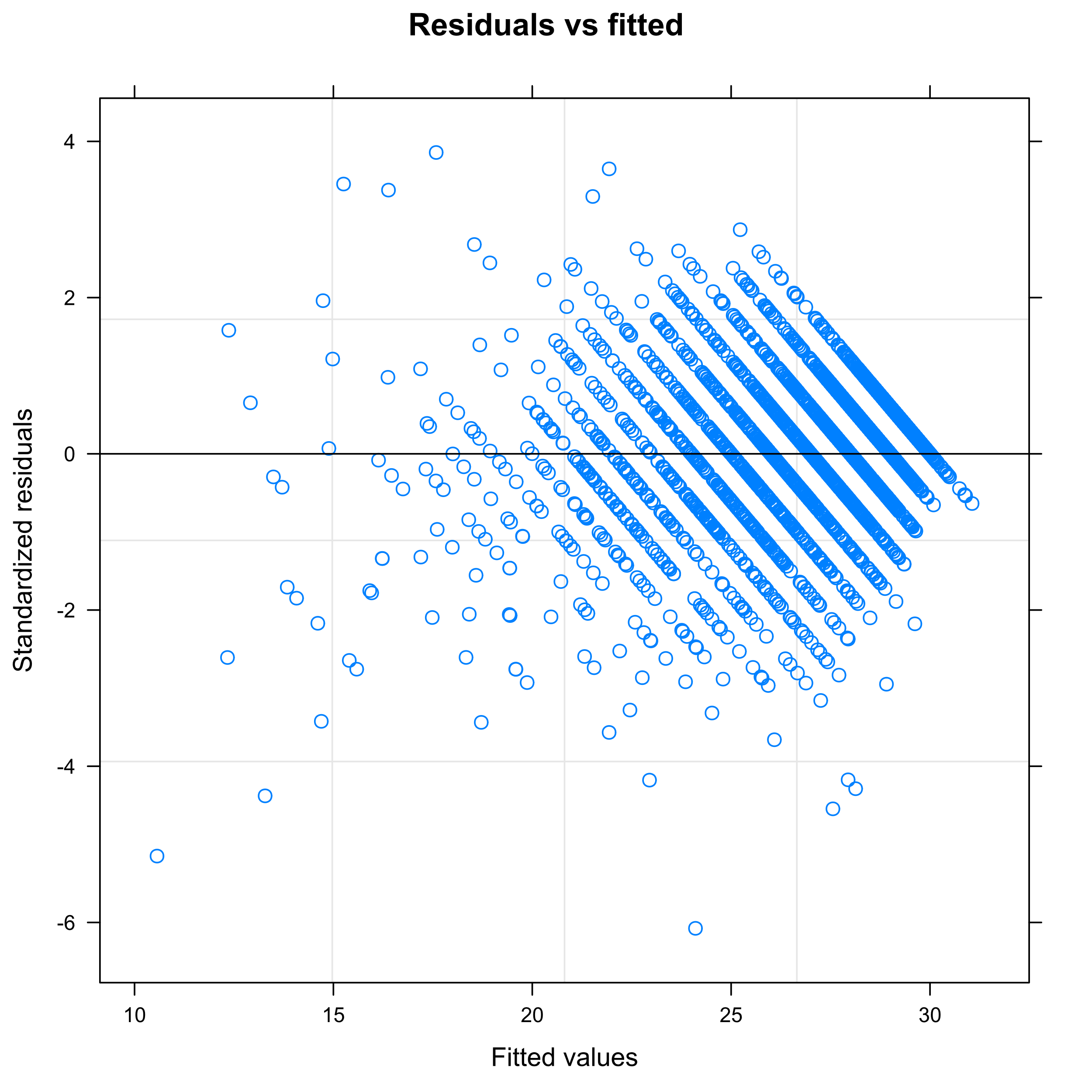

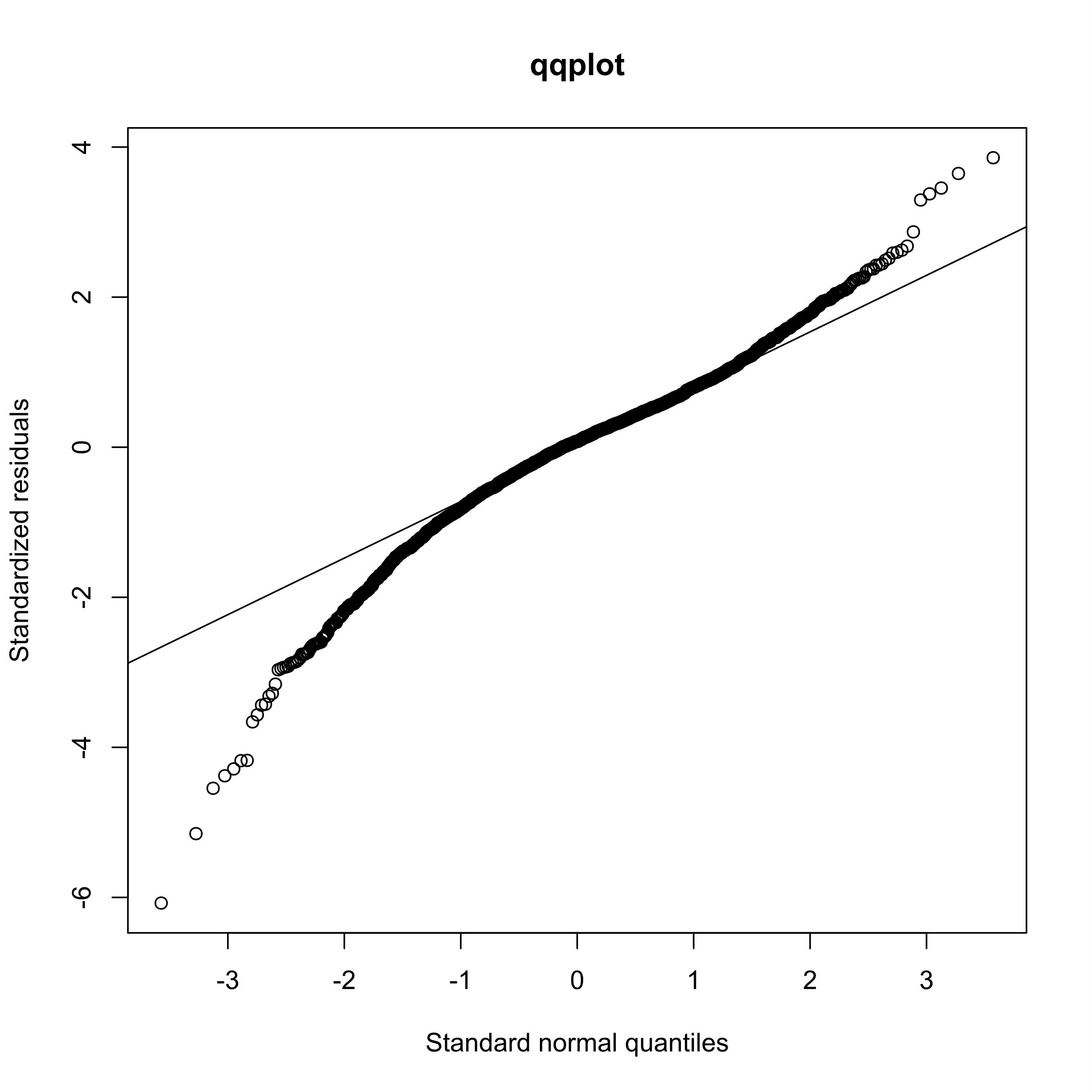

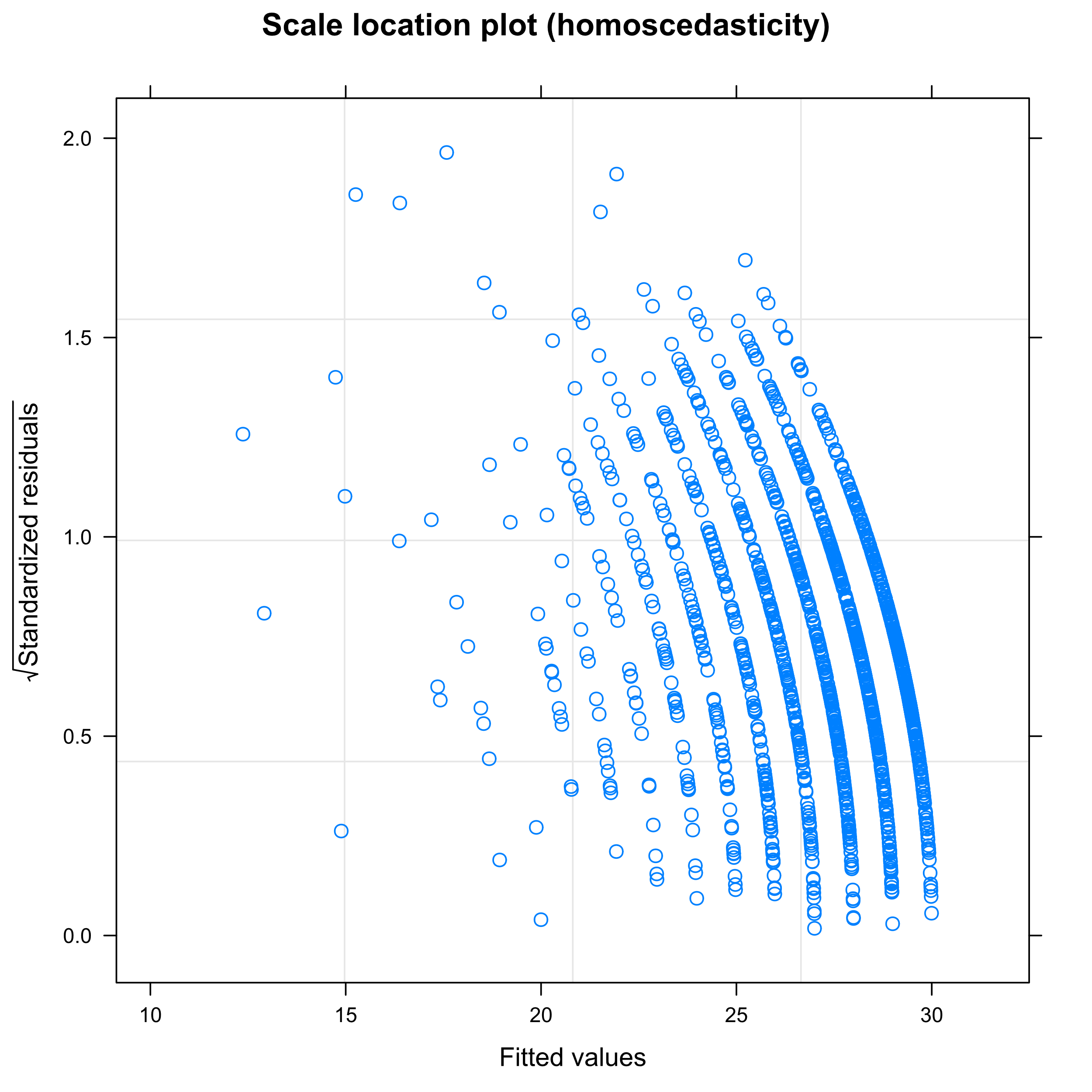


## Figure 7s: Examples of diagnostic plots for models based on the SDMT data from PD subjects in the PPMI study.


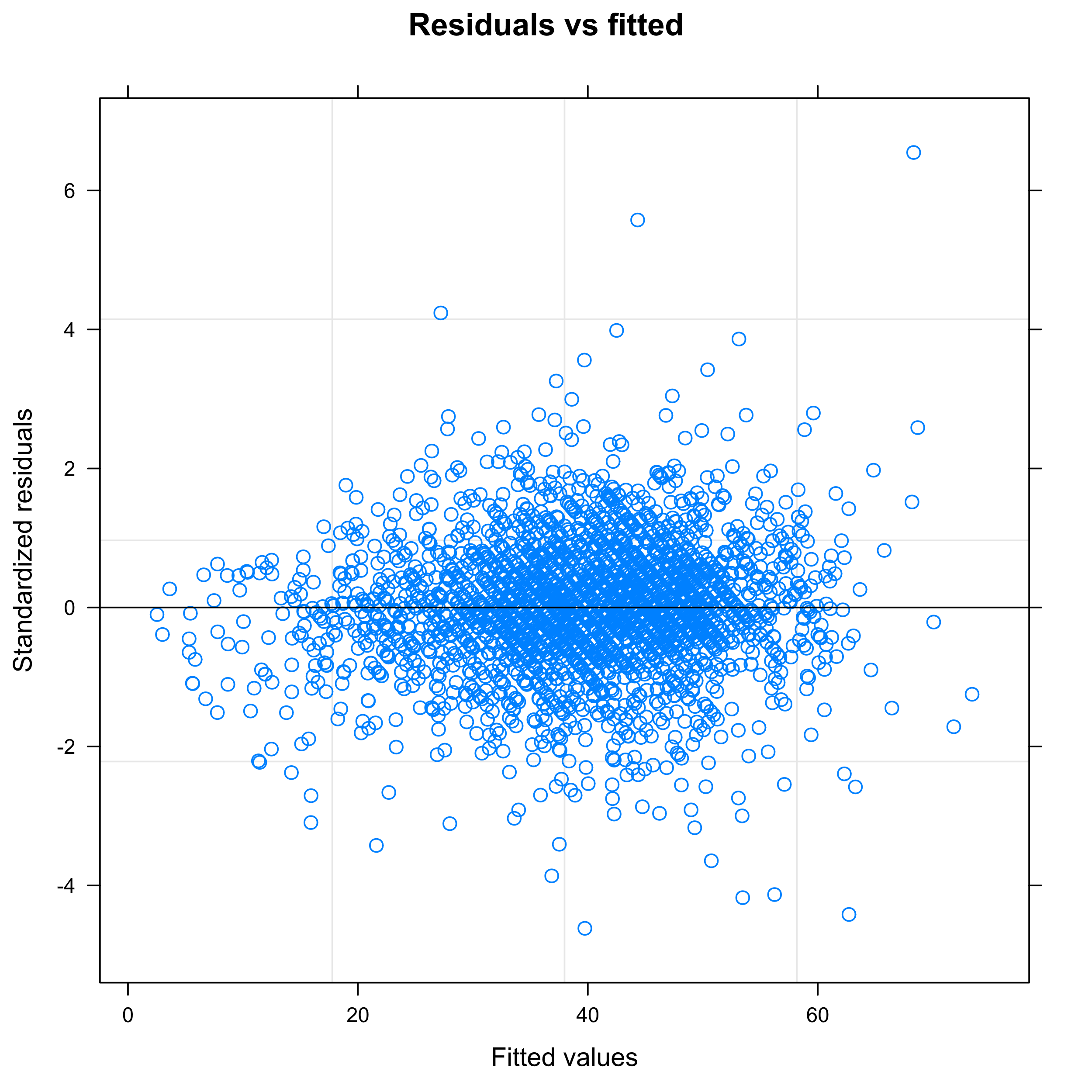

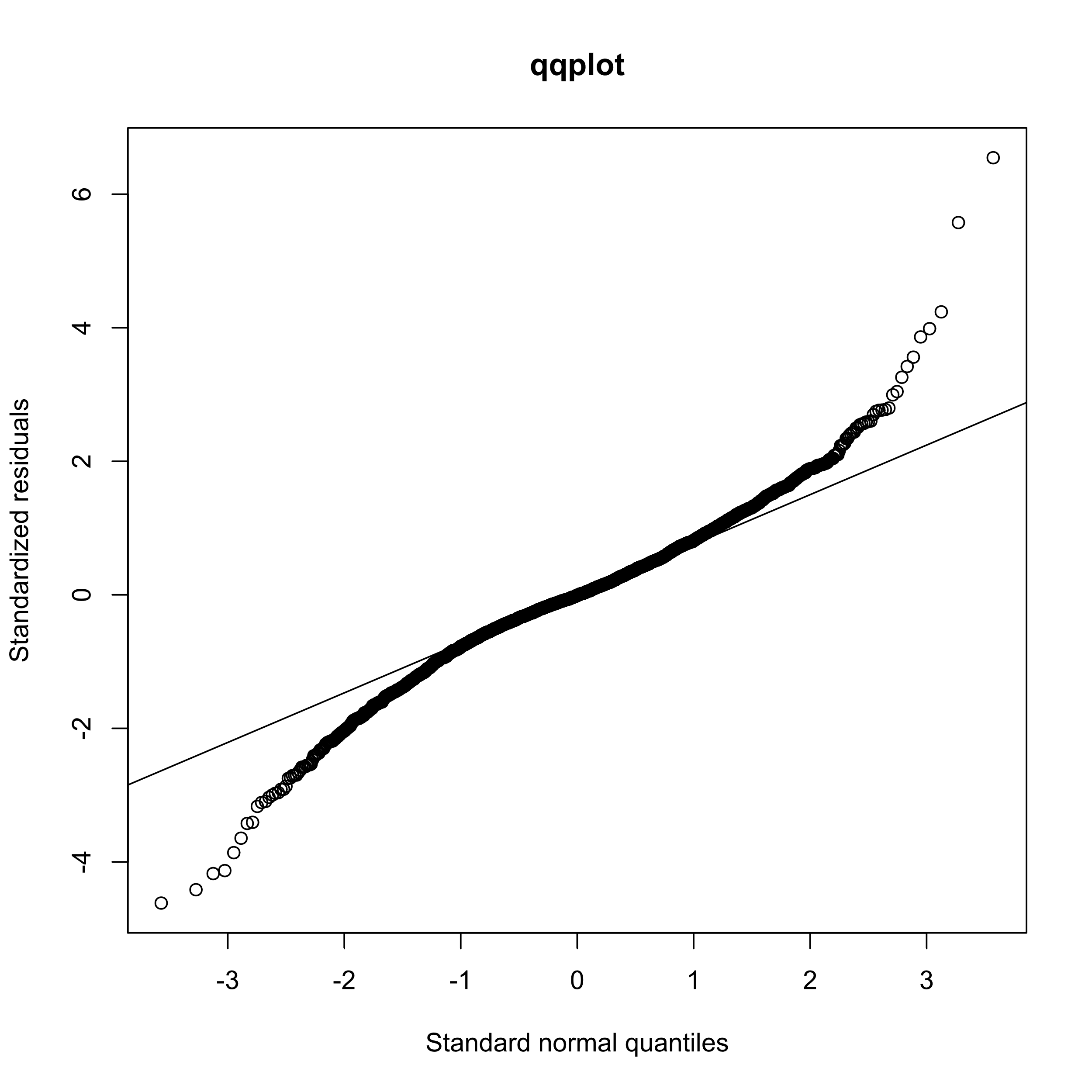

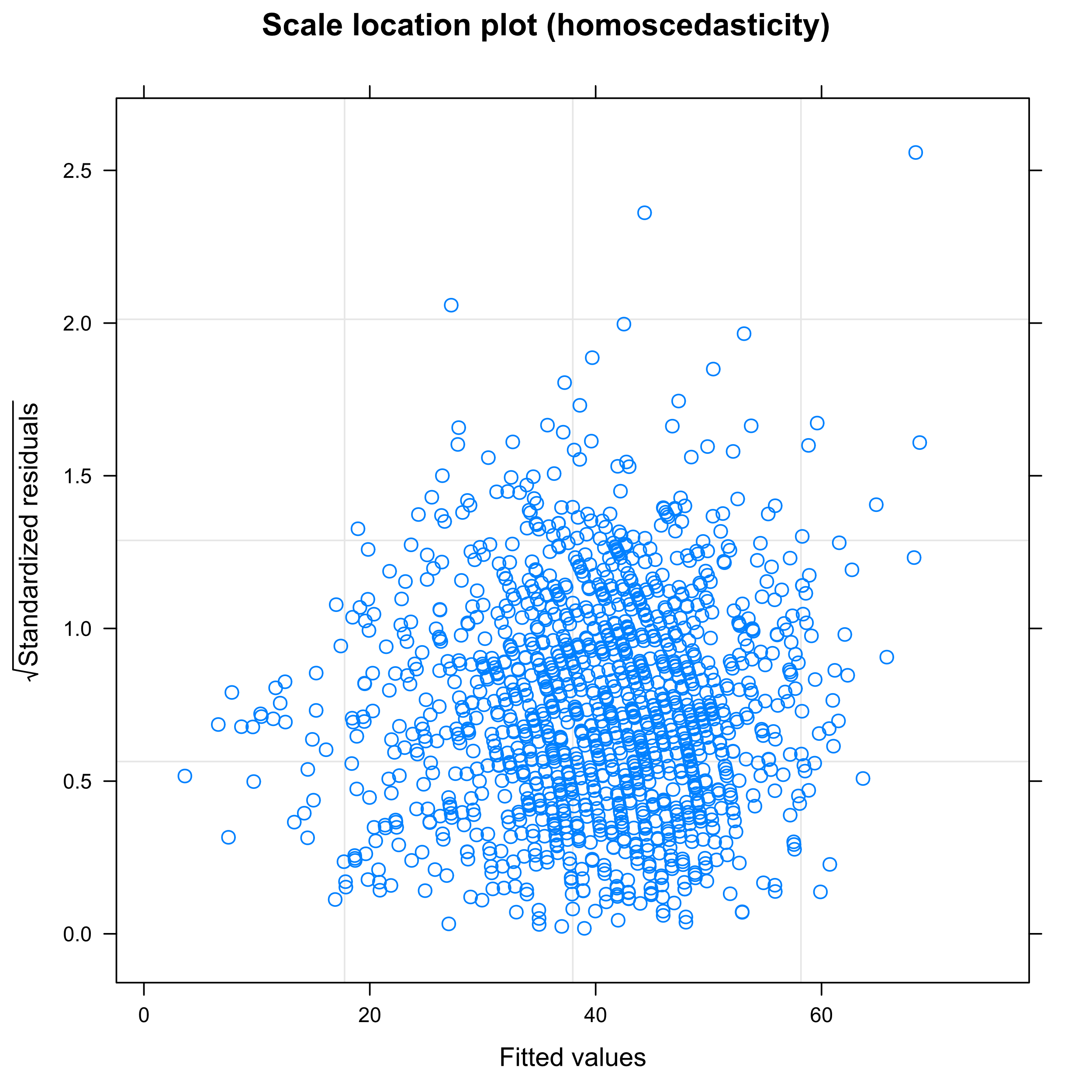


## Figure 8s: Examples of diagnostic plots for models based on the LNS data from PD subjects in the PPMI study.


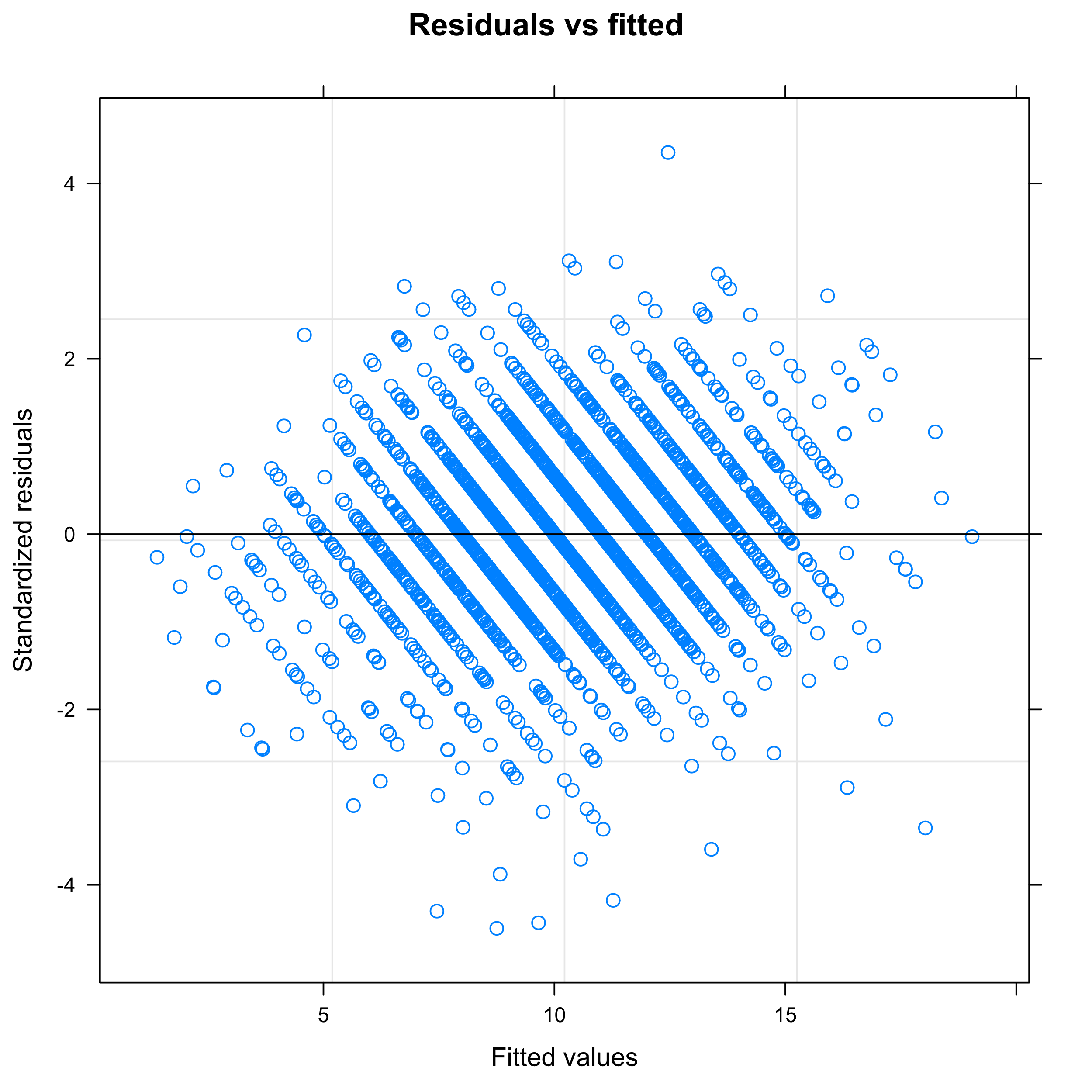

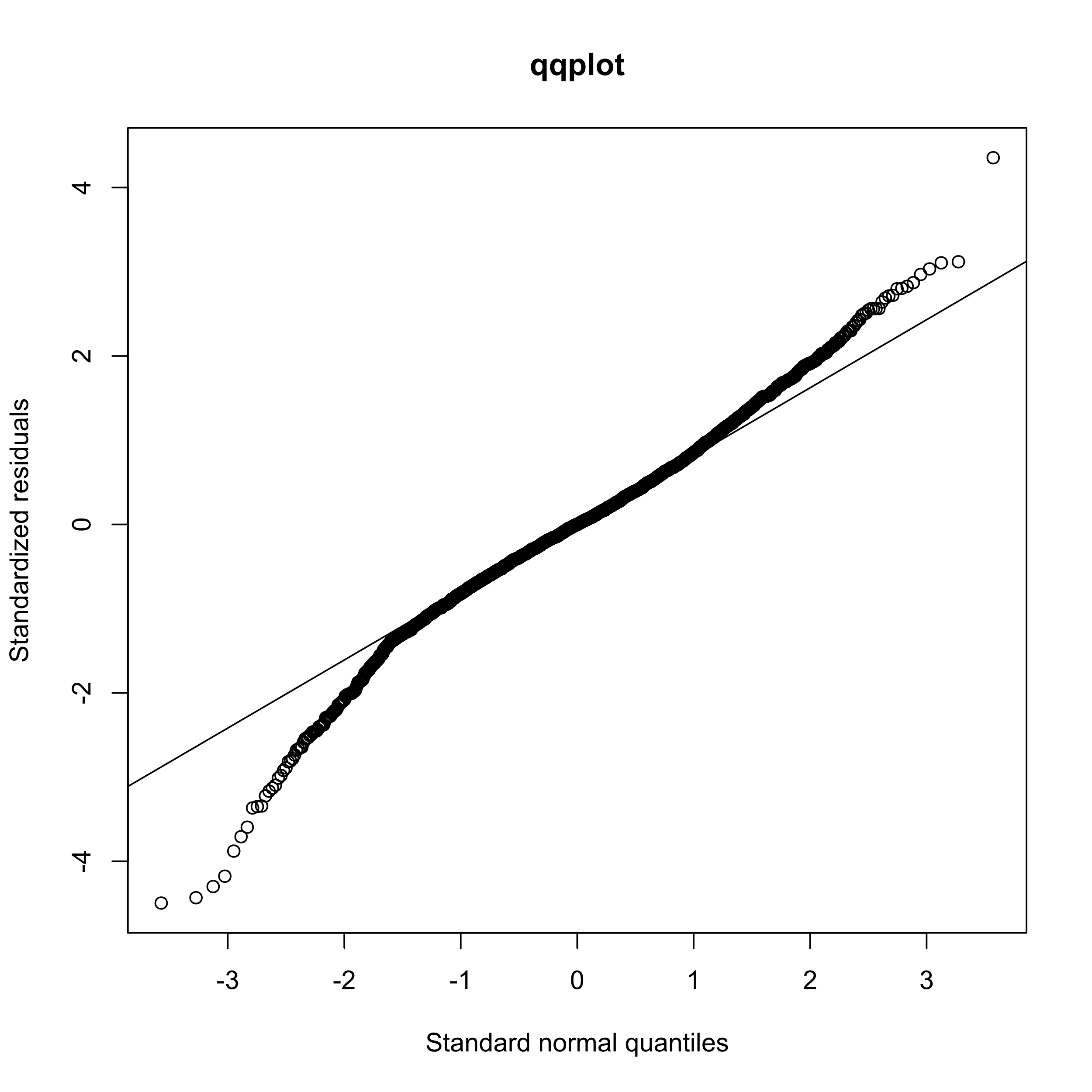

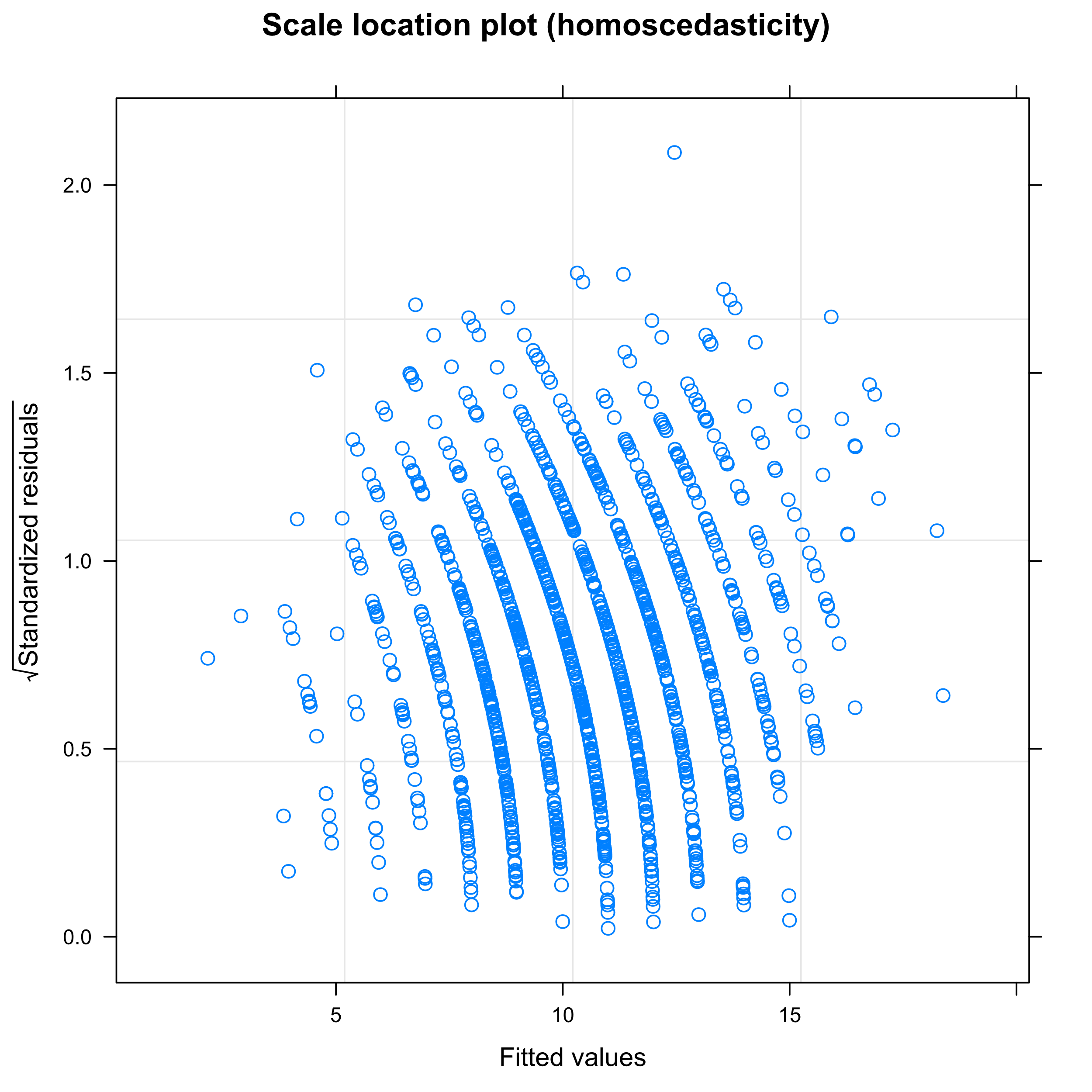


## Replication Chahine et al. (2019) study

One of the most important results Chahine et al. (2019) report is a greater annual rate of change in MoCA scores in PD patients with higher vascular risk scores (beta = -0.040, 95% CI = [-0.069 -- -0.011], p = 0.007) (reported in table 5 of their paper) (13). They used the 141 PD patients with an adequate MRI scan from PPMI in their analyses. Unfortunately, the exact subjects used in their final analysis are unknown to us, so we tried replicating it on all PD subjects, with the assessment of the relevant variables, included in the PPMI dataset (403 subjects). The linear mixed model they used for the results in table 5 is described in more detail in the online supplement of their paper. However, this supplement does not provide all details of the model either, so we assume the default structures for $\mathcal{R}_{\mathcal{i}}$ and $\mathcal{D}$ in the lme4 package (16).

Table 6s: replication of the model Chahine et al. used for the results in table 5 of their paper. Dependent

variable was the MoCA score. Exposure of interest in bold. mFrs: modified Framingham risk score, PD: Parkinson’s disease, MoCA: Montreal Cognitive Assessment

|  | **estimate** | **95% CI** | **p** |
| --- | --- | --- | --- |
| Intercept | 4.24 | 1.78 – 6.7 | 0.001 |
| mFRS | 0.0565 | -0.00686 – 0.12 | 0.083 |
| Year | 0.153 | 0.0478 – 0.259 | 0.004 |
| Age | -0.0349 | -0.0624 – -0.0073 | 0.014 |
| Male | -0.0154 | -0.366 – 0.335 | 0.932 |
| MoCA score BL | 0.834 | 0.772 – 0.895 | <0.0001 |
| PD duration BL | 0.18 | -0.109 – 0.469 | 0.226 |
| Education in years | 0.1 | 0.0442 – 0.156 | 0.001 |
| **Year:mFRS** | -0.0151 | -0.0228 – -0.00742 | 0.0001 |

We indeed obtain a comparable result in this replication attempt on the full PPMI dataset (table 6s).

However, Chahine et al. only correct the intercepts for confounding by age for example. If we take into

account that both faster cognitive decline and vascular risk is associated with higher age, the exposure of interest, year:mFRS, ceases to be statistically significant; a considerable part of the association between vascular risk and cognitive decline in PD patients can be explained by faster rate of change because of age (table 7s).

Table 7s: The model Chahine et al. used for the results in table 5 of their paper, but now taking faster cognitive decline (and PD progression) because of age into account. Dependent variable was the MoCA score. Exposure of interest in bold. mFrs: modified Framingham risk score, PD: Parkinson’s disease, MoCA: Montreal Cognitive Assessment

|  | **estimate** | **95% CI** | **p** |
| --- | --- | --- | --- |
| Intercept | 3.46 | 0.839 – 6.08 | 0.0104 |
| mFRS | 0.0257 | -0.0472 – 0.0987 | 0.492 |
| Year | 0.358 | 0.0951 – 0.621 | 0.00763 |
| Age | -0.0158 | -0.0513 – 0.0196 | 0.384 |
| Male | -0.012 | -0.363 – 0.339 | 0.947 |
| MoCA score BL | 0.834 | 0.772 – 0.896 | <0.0001 |
| PD duration BL | 0.179 | -0.11 – 0.468 | 0.229 |
| Education in years | 0.0998 | 0.0439 – 0.156 | 0.000565 |
| **Year:mFRS** | -0.00685 | -0.0193 – 0.00555 | 0.28 |
| Year:age | -0.00512 | -0.0111 – 0.000904 | 0.0956 |

## References

1. Jones J, Malaty I, Price C, Okun M, Bowers D. Health comorbidities and cognition in 1948 patients with idiopathic Parkinson’s disease. Parkinsonism & related disorders. 2012;18(10):1073–1078.

2. Jones JD, Jacobson C, Murphy M, Price C, Okun MS, Bowers D. Influence of hypertension on neurocognitive domains in nondemented Parkinson’s disease patients. Parkinson’s Disease. 2014;2014.

3. Jones JD, Tanner JJ, Okun M, Price CC, Bowers D. Are Parkinson’s patients more vulnerable to the effects of cardiovascular risk: a neuroimaging and neuropsychological study. Journal of the International Neuropsychological Society. 2017;23(4):322–331.

4. Swallow DM, Lawton MA, Grosset KA, Malek N, Klein J, Baig F, et al. Statins are underused in recent-onset Parkinson’s disease with increased vascular risk: findings from the UK Tracking Parkinson’s and Oxford Parkinson’s Disease Centre (OPDC) discovery cohorts. J Neurol Neurosurg Psychiatry. 2016;87(11):1183–1190.

5. Malek N, Lawton MA, Swallow DM, Grosset KA, Marrinan SL, Bajaj N, et al. Vascular disease and vascular risk factors in relation to motor features and cognition in early Parkinson’s disease. Movement Disorders. 2016;31(10):1518–1526.

6. Kotagal V, Albin RL, Müller ML, Koeppe RA, Frey KA, Bohnen NI. Modifiable cardiovascular risk factors and axial motor impairments in Parkinson disease. Neurology. 2014;82(17):1514–1520.

7. Kotagal V, Albin RL, Müller ML, Koeppe RA, Studenski S, Frey KA, et al. Advanced age, cardiovascular risk burden, and timed up and go test performance in Parkinson disease. Journals of Gerontology Series A: Biomedical Sciences and Medical Sciences. 2014;69(12):1569–1575.

8. Papapetropoulos S, Ellul J, Argyriou A, Talelli P, Chroni E, Papapetropoulos T. The effect of vascular disease on late onset Parkinson’s disease. European Journal of Neurology. 2004;11(4):231–235.

9. Sterling NW, Lichtenstein M, Lee E-Y, Lewis MM, Evans A, Eslinger PJ, et al. Higher plasma LDL-cholesterol is associated with preserved executive and fine motor functions in Parkinson’s disease. Aging and Disease. 2016;7(3):237.

10. Huang X, Auinger P, Eberly S, Oakes D, Schwarzschild M, Ascherio A, et al. Serum cholesterol and the progression of Parkinson’s disease: results from DATATOP. PLoS One. 2011;6(8):e22854.

11. Yoo HS, Chung SJ, Lee PH, Sohn YH, Kang SY. The Influence of Body Mass Index at Diagnosis on Cognitive Decline in Parkinson’s Disease. Journal of Clinical Neurology. 2019;15(4):517–526.

12. Kim R, Jun J-S. Impact of Overweight and Obesity on Functional and Clinical Outcomes of Early Parkinson’s Disease. Journal of the American Medical Directors Association. 2020;

13. Chahine L, Dos Santos C, Fullard M, Scordia C, Weintraub D, Erus G, et al. Modifiable vascular risk factors, white matter disease and cognition in early Parkinson’s disease. European Journal of Neurology. 2019;26(2):246–e18.

14. Doiron M, Langlois M, Dupré N, Simard M. The influence of vascular risk factors on cognitive function in early Parkinson’s disease. International Journal of Geriatric Psychiatry. 2018;33(2):288–297.

15. Mollenhauer B, Zimmermann J, Sixel-Döring F, Focke NK, Wicke T, Ebentheuer J, et al. Baseline predictors for progression 4 years after Parkinson’s disease diagnosis in the De Novo Parkinson Cohort (DeNoPa). Movement Disorders. 2019;34(1):67–77.

16. Bates D, Mächler M, Bolker B, Walker S. Fitting Linear Mixed-Effects Models Using lme4. Journal of Statistical Software. 2015;67(1):1–48.
